# Polychlorinated biphenyls (PCBs) interact with drug metabolism in vivo

**DOI:** 10.1007/s00204-025-04145-6

**Published:** 2025-08-15

**Authors:** Julian Peter Müller, Jens Rengelshausen, Salah Laieb, Maryam Safavi, Andrea Kaifie, Andre Esser, Thomas Schettgen, Jens Bertram, Julia Krabbe, Elke Schaeffeler, Jens Sarömba, Katja S. Just, Roman Tremmel, Matthias Schwab, Julia C. Stingl, Thomas Kraus, Patrick Ziegler

**Affiliations:** 1 Institute of Clinical Pharmacology, University Hospital of RWTH, 52074 Aachen, Germany; 2 Institute for Occupational, Social and Environmental Medicine, Medical Faculty, RWTH Aachen University, Pauwelsstrasse 30, 52074 Aachen, Germany; 3 Dr. Margarete Fischer-Bosch-Institute of Clinical Pharmacology, Stuttgart and University of Tuebingen, Tuebingen, Germany; 4 Departments of Clinical Pharmacology, and Pharmacy and Biochemistry, University of Tuebingen, Tuebingen, Germany; 5 Department of Clinical Pharmacology and Pharmacoepidemiology, University Hospital Heidelberg, Heidelberg, Germany

**Keywords:** Cytochrome P450, Polychlorinated biphenyls (PCBs), Drug metabolism, Environmental toxicology, CYP1A2/CYP2C9 enzyme modulation

## Abstract

Polychlorinated biphenyls (PCBs) are widespread environmental contaminants that interfere with xenobiotic metabolism, primarily by modulating cytochrome P450 (CYP) enzymes. However, their pharmacokinetic consequences in exposed individuals remain poorly defined. Here, we investigated the impact of PCB exposure on CYP enzyme activity using a combined clinical pharmacokinetic and in vitro mechanistic approach. Ten occupationally PCB-exposed individuals from the German HELPcB cohort and ten controls received a CYP phenotyping cocktail to assess enzyme function (Clinical Trial Registry: DRKS00028922). Plasma drug and metabolite concentrations were quantified to evaluate CYP1A2, CYP2B6, CYP2C9, CYP2C19, CYP2D6, and CYP3A activity, considering genetically defined metabolizer status. PCB118 exposure was significantly associated with reduced CYP1A2 activity (R^2^ = 0.155, t = − 2.115, p = 0.049, β = − 0.446), indicating a decrease in CYP1A2 activity with higher PCB118 levels. This was further supported by in vitro assays demonstrating dose-dependent inhibition. In addition, PCB74 exposure showed a trend toward increased CYP2C9 activity, suggesting a potential inductive effect. Mechanistic studies revealed that PCB118 acts as both an inhibitor and a substrate of CYP1A2, generating reactive arene oxide intermediates that may contribute to mechanism-based inhibition. These findings challenge the traditional view of PCBs as merely chronic toxicants, showing that they can acutely alter drug metabolism and potentially impact drug efficacy and safety. Given the widespread presence of PCBs and other persistent organic pollutants, these results highlight the need to integrate environmental toxicant exposure into pharmacokinetic models to optimize drug therapy and minimize adverse effects.

**Supplementary Information:**

The online version contains supplementary material available at 10.1007/s00204-025-04145-6.

## Introduction

Drug metabolism is a highly dynamic and multifaceted process influenced by both intrinsic and extrinsic factors. Among these, the cytochrome P450 (CYP) enzyme superfamily plays a central role in metabolizing pharmaceuticals and environmental contaminants (Zanger and Schwab 2013). Genetic polymorphisms within CYP genes contribute substantially to interindividual variability in drug metabolism, affecting drug clearance, efficacy, and toxicity (Zanger and Schwab 2013). While genetic factors, which account for 20–95% of interindividual differences in drug metabolism, remain constant throughout life (Kalow et al. 1998), their impact is dependent on the specific CYP enzyme involved. In addition, extrinsic influences such as diet, co-medications, and environmental exposures can dynamically modulate CYP activity in a transient and reversible manner (Kantola et al. 1998; Ruschitzka et al. 2000; Gareri et al. 2008). In addition, disease states such as hepatic dysfunction or systemic inflammation can further alter CYP activity, compounding pharmacokinetic variability (Fisher et al. 2009; Kawashima et al. 2023; Lenoir et al. 2021; Morgan 2009).

Unlike the documented in vivo effects of diet and medications on CYP activity in humans, the impact of environmental contaminants remains less explored in clinical settings. Most studies have relied on in vitro experiments, animal models, or noninvasive human biomaterial analyses (Stoddard et al. 2021; Lee and Yang 2008). In these experimental systems, environmental pollutants such as polycyclic aromatic hydrocarbons (PAHs) have been shown to induce CYP enzymes through activation of the aryl hydrocarbon receptor (AhR), leading to increased expression of the CYP1A and CYP3A subfamilies (Stoddard et al. 2021).

Polychlorinated biphenyls (PCBs), another group of widespread environmental contaminants, enter the human body primarily through food, skin contact, and inhalation (Lauby-Secretan et al. 2013). Studies in cell lines and experimental animals have demonstrated that PCB congeners can both induce and inhibit human CYP enzyme activity (Pang et al. 1999; Maron and Ames 1983). Moreover, there is increasing evidence that certain PCBs are not only inducers but also substrates of CYP enzymes, raising concerns about their potential to compete with drugs for CYP-mediated metabolism (Matsusue et al. 1996; McGraw and Waller 2006; Uwimana et al. 2018; Randerath et al. 2024). Similar inhibitory interactions have been observed with industrial solvents such as toluene, which competes with paracetamol for CYP1A2 metabolism (Lof et al. 1990). However, the clinical significance of such interactions remains largely unexplored.

Given the long biological half-life of PCBs and their bioaccumulation in exposed populations (Esser et al. 2021), it is tempting to speculate whether chronic PCB exposure alters drug metabolism and pharmacokinetics. This study aims to bridge this knowledge gap by systematically investigating the impact of PCB exposure on CYP enzyme activity both in vivo and in vitro. Using a clinical pharmacokinetic approach, we employ a cocktail study design, where multiple CYP-specific probe drugs are administered simultaneously to evaluate enzyme activity in PCB-exposed individuals. This approach allows for precise assessment of CYP function without significant drug–drug interactions (Donzelli et al. 2014; Lenoir et al. 2021; Matthaei et al. 2015).

Complementary in vitro studies will further explore whether specific PCB congeners act as competitive inhibitors or inducers of CYP enzymes, potentially interfering with the metabolism of drug substrates. By integrating these findings, this research aims to determine the existence of PCB–drug interactions and, if present, to elucidate their underlying mechanisms. The results will contribute to a better understanding of PCB-related risks in pharmacotherapy and aid in improving drug safety in exposed populations.

## Materials and methods

### Clinical study

The clinical study was approved by the Ethics Committee of the Medical Faculty of RWTH Aachen, was conducted at the University Hospital Aachen in accordance with the Declaration of Helsinki, and registered in the German Clinical Trials Register (DRKS00028922).

### Study population

Ten adult subjects with prior occupational exposure to PCBs from the German HELPcB cohort and ten control subjects who were not knowingly PCB exposed participated in the study after they had been fully informed about the study and given written informed consent. The participants were ascertained not to have any acute disease by medical history, physical examination, laboratory screening, including hematological and biochemical blood tests, and a 12-lead electrocardiogram. None of the subjects had a chronic disease or a chronic medication that is known to interfere with CYP mediated drug metabolism except for one subject who was on atorvastatin medication. The demographic data of the subjects are summarized in Table 1 in the supplemental data. At the enrollment visit, 2 blood samples (9 ml each) were collected into ethylenediaminetetraacetic acid (EDTA)-containing tubes taken from each subject for analyzing the plasma concentrations of PCB congeners and for pharmacogenetic analysis.

### Study design and procedures

According to a controlled, open-label, fixed dose schedule design, each participant received a single oral dose of a drug cocktail designed for CYP phenotyping, including 50 mg caffeine (for CYP1A2 and CYP2A6), 50 mg efavirenz (for CYP2B6), 2.5 mg torsemide (for CYP2C9), 10 mg omeprazole (for CYP2C19), and 12.5 mg metoprolol (for CYP2D6), and 1 mg midazolam (for CYP3A). Three subjects did not receive efavirenz for technical reasons. Participants stayed fasted from the evening before until 3 h after drug administration. After that, a meal was provided. Water and juice were permitted but alcoholic and caffeinated beverages were not allowed from the evening before drug administration until the last blood sample was taken on the next day. Venous blood samples (9 ml each) were collected through an intravenous catheter immediately before and 0.25, 0.5, 0.75, 1, 1.5, 2, 2.5, 3, 4, 5, 6, 7, 8, and 24 h after drug administration. Blood was drawn into EDTA-coated tubes, centrifuged at 1500 g for 10 min at 4 °C, and plasma samples were subsequently stored at − 80 °C until further analysis. After completely voiding the bladder immediately before drug administration, the participants collected urine from 0 to 24 h after drug administration. After weighing the collected urine, a 10 ml aliquot of each fraction was taken.

### Pharmacogenetic analysis

Genotyping for common SNPs and respective diplotypes in the genes of CYP1A2 and CYP2A6 was performed as described by Randerath et al. (2024), while genotyping for CYP2B6, CYP2C9, CYP2C19, CYP2D6, and CYP3A4/5 followed the methodology outlined by Müller et al. (2024). In brief, CYP1A2 and CYP2A6 genotypes were extracted from SNP array data (Infinium™ Global Screening Arrays (GSA)-v2.0 (Illumina Inc.)) using pypgx 0.19.0 and the chip pipeline with the impute option and 1000 Genomes Project (1KGP) data as reference. Array experiments were performed by LIFE and BRAIN GmbH, Bonn, Germany. The genotypes of the remaining genes were characterized using a TaqMan OpenArray panel (ThermoFisher, Waltham, MA) measured at the IKP Stuttgart and results were 100% in concordance with the SNP array results. In addition, *CYP2A6* and *CYP2D6* deletions and duplications were determined using TaqMan copy number assays Hs07545274_cn and Hs00010001_cn, respectively. The genotypes were translated into metabolizer phenotypes according to de Jong et al. (2023) and according to CPIC (CPIC® Guideline for Opioids and CYP2D6, OPRM1, and COMT. < https://cpicpgx.org/guidelines/guideline-for-codeine-andcyp2d6/ > (2023). Accessed August 15, 2023).

### Determination of PCB concentrations in plasma

Plasma PCB concentrations of 19 PCB congeners (PCB 28, PCB 52, PCB 101, PCB 153, PCB 138, PCB 180, PCB 81, PCB 77, PCB 123, PCB 118, PCB 114, PCB 105, PCB 126, PCB 167, PCB 156, PCB 157, PCB 169, PCB 189) were quantified using a previously validated liquid chromatography–mass spectrometry (LC–MS) method (Schettgen et al. 2012). The laboratory regularly participates in round robins for the determination of PCBs in plasma (www.g-equas.de).

### Probe drug and metabolite quantification

Commercially sourced standards and internal standards were used for quantification (see Supplemental Materials). In brief, 20 µl of the plasma samples were protein-precipitated with 80 µl methanol containing all internal standards or were treated for 16 h at 37 °C with 2 µl β-glucuronidase (1,000,000 Units) for deconjugation before protein precipitation with 78 µl methanol containing internal standards. Samples were centrifuged for 20 min, at 4 °C and 17,000 × *g*, and 40 µl of the supernatants were diluted with 40 µl LC–MS grade water, centrifuged again for 5 min at 4 °C and 17,000 × *g* and 50 µl supernatant were transferred to LC–MS vials 5 µl of the samples were injected into the LC–MS system.

### LC–MS analysis

An Agilent 1290 Infinity II UHPLC coupled with a SCIEX QTRAP6500 + triple quadrupole mass spectrometer was used. Chromatographic separation was achieved on an Agilent Poroshell 120 EC-C18 (1.9 µm, 2.1 × 50 mm) column with A: 0.1% formic acid in water and B: methanol with a flow rate of 0.7 ml/min and a total run time of 8.5 min. Analytes were eluted using a gradient program. The following gradient steps represent gradual changes in % B over time: 5% B from 0 to 0.2 min, 30% B at 2 min, 34% B at 4.8 min, 95% B at 6.5 min, 95% B at 7.5 min and re-equilibrating to 5% B at 7.7 min and 5%B till 8.5 min. A representative chromatogram is shown in supplemental Fig. 2. The scheduled MRM mode was applied for analyte detection in both positive and negative ESI modes (Supplementary Table 4). Calibration curves were constructed with linear regression using weighted (1/x^2^) models (detailed information in supplemental methods).

### Validation of the analytical method

The method was validated for intra- and interday accuracy and precision, stability, and matrix effects. Accuracy and precision, stability and matrix effects for the quantification of probe drugs and metabolites were within acceptable ranges, with coefficients of variation ≤ 15% and mean accuracy between 85 and 115% (or 80–120% at the LLOQ) with few exceptions. Validation details are provided in Supplementary Tables 5–7 and supplemental Fig. 3. Glucuronidase/sulfatase digests were used to isolate CYP-specific metabolic activities, reducing interference from polymorphic UDP-glucuronosyltransferase (UGT) and sulfotransferase isoforms.

### Pharmacokinetic analysis

Noncompartmental analysis using Phoenix WinNonlin 8.4.0.6172 software (Certara, Radnor, PA, U.S.A.) was performed to determine pharmacokinetic parameters of caffeine, paraxanthine, efavirenz, 8-OH-efavirenz, torsemide, OH-torsemide, omeprazole, 5-OH-omeprazole, metoprolol, α-OH-metoprolol, midazolam and 1-OH-midazolam. Partial areas under the plasma concentration–time curves from 0 to 3 h (AUC_0–3 h_), from 0 to 4 h (AUC_0–4 h_), and from 0 to 8 h (AUC_0–8 h_) were calculated based on measured concentrations by the linear trapezoidal rule without extrapolation. Concentration data from the 24-h sampling timepoint were below the LLOQ for some compounds and their metabolites in several subjects. Therefore, AUC_0–24 h_ values were not calculated to avoid extrapolations and to ensure comparability of data between subjects for statistical analysis. Metabolic ratios (MR) were calculated by dividing the partial AUC of the metabolite by the respective partial AUC of the parent drug. The MRs were used as biomarkers for the individual CYP activities of the study subjects.

### In vitro metabolism of PCB118 by CYP1A2 Supersomes™

To investigate the metabolism of PCB118 by CYP1A2, incubation assays were conducted using CYP1A2 supersomes in a total reaction volume of 100 µL. Reaction mixtures were assembled on ice. Each reaction contained 50 mM potassium phosphate buffer (pH 7.4), 50 pmol/mL CYP1A2 supersomes, 2 mM NADPH, and 10 µM of the respective substrate, including caffeine, phenacetin, PCB28, or PCB118. Reactions were initiated by the addition of CYP1A2 supersomes. Incubations were carried out at 37 °C with shaking at 250 rpm, with reaction times of 1 h for caffeine, phenacetin, PCB28, and PCB118, and additional 4 h for PCB28 and PCB118.

To assess potential non-enzymatic transformations and confirm NADPH-dependent metabolism, control incubations were performed under the following conditions: buffer and enzyme only (no-substrate control), substrate, buffer, and NADPH without enzyme (no-enzyme control), and substrate, buffer, and enzyme without NADPH (no-NADPH control). Each experimental condition was conducted in triplicate, while control conditions were performed as single replicates.

For the detection of caffeine and phenacetin metabolites, reactions were terminated by the addition of 400 µL methanol containing internal standards, followed by vortexing for 10 s and centrifugation at 21,300 × *g* for 30 min at 4 °C. The resulting supernatants (100 µL) were transferred to LC–MS vials, yielding samples with 80% methanol content for analysis. For the detection of hydroxylated PCB metabolites, reactions were stopped by adding 500 µL methanol, followed by the addition of 50 µL of an internal standard mix (10 ng/mL). Samples were vortexed for 1 min and centrifuged at 4,500 rpm for 10 min. The resulting supernatants were transferred to glass LC vials and evaporated under a nitrogen stream at 45 °C until approximately 50 µL remained. Samples were then reconstituted in 100 µL of 0.1 M ammonium acetate buffer (pH 5.3) before transfer to inserts for LC–MS analysis.

### CYP1A2 and CYP2C9 activity assessment via luminescent assays, transgenic HEK293 cells, and primary human hepatocytes

CYP1A2 and CYP2C9 enzyme activity was first assessed using a luciferase-based P450-Glo™ screening system (Promega, Madison, WI, USA) following the manufacturer’s instructions. The reaction mixture consisted of 1 nM recombinant enzyme (CYP1A2 or CYP2C9), 250 µM luciferin–ME substrate, 0.1 M potassium phosphate buffer (pH 7.4), and an NADPH regeneration system (including NADP⁺, glucose-6-phosphate, MgCl₂, and glucose-6-phosphate dehydrogenase), in a total volume of 50 µL. Controls for enzyme inhibition included α-naphthoflavone (1 µM) for CYP1A2 and sulfaphenazole (2 µM) for CYP2C9. After preincubation at 37 °C for 10 min (excluding NADPH), the reaction was initiated by adding the NADPH regeneration system and incubated for 30 min. To terminate the reaction and generate luminescence, 50 µL of Luciferin Detection Reagent was added, followed by a 20-min incubation at room temperature. Luminescence was measured using a luminometer, with light intensity proportional to CYP activity. The assay tolerated up to 0.25% DMSO without significant inhibition. Enzyme activity was quantified relative to a d-luciferin standard curve and expressed as pmol luciferin/min/pmol CYP. In addition we evaluated the effects of PCB74 and PCB118 on CYP1A2 and CYP2C9 activity in HEK293 cells stably overexpressing the respective enzymes. A total of 500,000 cells were seeded onto 6-well plates in DMEM medium and treated with 0.1, 0.5, or 1 µM of PCB74 (for CYP2C9) or PCB118 (for CYP1A2), alongside vehicle controls. Cells were maintained at 37 °C with 5% CO₂ throughout the experiment. For CYP2C9 activity assays, cells were incubated with PCB74 for 24 h, followed by the addition of 10 µM torsemide and further incubation for 24 h before metabolite analysis. For CYP1A2 activity assays, cells were incubated with PCB118 for 30 min, followed by the addition of 10 µM phenacetin and an additional 1-h incubation. After treatment, 50 µL of DMEM supernatant was subjected to protein precipitation using 200 µL methanol containing internal standards (OH-torsemide-d7 or paracetamol-d3), followed by centrifugation at 17,000 × g for 30 min at 4 °C. Supernatants were transferred to LC–MS vials and analyzed for relevant metabolites. Details on metabolite quantification are provided in the supplemental file. In addition, primary human hepatocytes were used to assess transcriptional responses to PCB118 and PCB74. Hepatocytes were obtained from Thermo Fisher Scientific (Waltham, MA, USA) and cultured according to the supplier’s protocol (background information on hepatocytes can be found in the supplement). To optimize seeding density, hepatocytes were first cultured in suspension at 1.5 × 10⁶ cells/mL. A 1 mL aliquot of this stock was diluted in 99 mL of pre-warmed plating medium, yielding a working concentration of 15,000 cells/mL. Wells of a 96-well plate were pre-wetted with 100 µL plating medium, followed by the addition of 100 µL of the working cell suspension, resulting in 1,500 cells per well (total volume: 200 µL). Plates were centrifuged at 200 × g for 2 min and incubated at 37 °C with 5% CO₂ for 5 days. On day 5, 100 µL of medium was replaced with fresh medium, and incubation continued for 2 more days. On day 7, 100 µL of the medium was removed and replaced with medium containing Omeprazole, PCB118, or PCB74. After 48 h of exposure, the contents of 16 wells per treatment group were pooled, centrifuged, and the supernatant discarded. Trizol reagent was added to the cell pellet for subsequent RNA extraction and gene expression analysis.

### RNA isolation and cDNA synthesis

Total RNA was isolated from primary human hepatocytes using TRIzol reagent. A total of 750 µL of TRIzol was added to the cell pellet and incubated at room temperature for 5 min, followed by the addition of 0.2 mL chloroform. Samples were vortexed and incubated for 3 min at room temperature, followed by centrifugation at 13,000 × *g* for 15 min at 4 °C. The upper aqueous phase (~ 600 µL) was carefully transferred to an RNase-free tube and kept on ice.

For RNA precipitation, 0.5 mL isopropanol was added, and samples were incubated on ice for 10 min before centrifugation at 12,000 × *g* for 10 min at room temperature. The supernatant was discarded, and the pellet was air-dried for approximately 20 min before being resuspended in 40 µL of RNase-free water. RNA was incubated at room temperature for 1 min and heated at 55 °C for 10 min before quantification using a NanoDrop spectrophotometer.

cDNA synthesis was carried out using the SuperScript™ III First-Strand Synthesis SuperMix (Thermo Fisher Scientific), following the manufacturer’s protocol with minor modifications. Reactions were assembled on ice, containing 10 µL of 2X RT Reaction Mix, 2 µL of RT Enzyme Mix, up to 1 µg of RNA, and DEPC-treated water to a final volume of 20 µL. The thermocycling program consisted of 25 °C for 10 min, 50 °C for 30 min, and 85 °C for 5 min, followed by cooling on ice. Residual RNA was removed by the addition of 1 µL (2 U) of *E. coli* RNase H, followed by incubation at 37 °C for 20 min. The synthesized cDNA was used immediately for qPCR or stored at − 20 °C.

### Quantitative real-time PCR (qRT-PCR) analysis

Gene expression analysis was performed using TaqMan® Fast Advanced Master Mix. Each reaction was prepared in a 96-well plate with a total volume of 20 µL, consisting of 10 µL of TaqMan® Fast Advanced Master Mix (2X), 1 µL of TaqMan® Assay primer/probe (20X), 4 µL of cDNA template (200 ng per well), and nuclease-free water. qPCR reactions were performed under the following conditions: UNG activation at 50 °C for 2 min, polymerase activation at 95 °C for 20 s, followed by 40 cycles of denaturation at 95 °C for 3 s and annealing/extension at 60 °C for 30 s. Amplifications were conducted according to the instrument manufacturer’s instructions.

### Statistical analysis

Data collection and handling were conducted using Microsoft Excel 2018 (Microsoft Corporation) and Microsoft Access 2018. All statistical analyses were performed using SAS 9.4 (SAS Institute Inc., 2013), and figures were generated using GraphPad Prism 10.4.1 (GraphPad Software, LLC).

To analyze differences in the metabolism rates of various substances in the cocktail study, the area under the curve (AUC) of metabolic ratios (MRs) over time was used as the primary outcome measure. Initially, linear regression models were used to evaluate raw associations between PCB exposure and CYP enzyme activities, as represented by metabolic ratios (Table 1). In these models, metabolic ratios were treated as dependent variables, while PCB plasma concentrations were considered independent variables.

A hierarchical generalized linear mixed model (HGLMM) was applied using the GLIMMIX procedure in SAS to account for repeated measures and inter-individual variability. PCB plasma concentrations (PCB74, PCB118, PCB138, and total PCB) were included as independent variables. The models were adjusted for smoking status and gender (categorical variables) as well as BMI and age (continuous variables) as potential confounders. Model fit was evaluated using the akaike information criterion (AIC), corrected AIC (AICC), Bayesian information criterion (BIC), and log-likelihood statistics, to identify the best adjustment for the distribution of the outcome variable. Degrees of freedom were adjusted for variance estimates using the Kenward–Roger 2 method (Faul et al. 2009).

For pharmacogenetic analyses, metabolizer status was determined for *CYP1A2, CYP2B6, CYP2C9, CYP2C19, CYP2D6, CYP3A4, and CYP3A5* based on genotyping data (Supplementary Table 3). The activity scores of CYP2D6 (for metoprolol) and CYP2C19 (for omeprazole) were included as additional continuous covariates in the models to account for genetic variability in drug metabolism.

For the in vitro experiments, statistical analyses were also conducted using the GLIMMIX procedure in SAS. A generalized linear mixed model (GLMM) was applied, using a lognormal response distribution and an identity link function. Treatment condition (PCB concentration) was included as a fixed effect, while the respective enzymatic activity or metabolite formation was treated as the dependent variable. Least-squares means (LS-Means) were calculated to compare the effects of different concentrations with the control group (0 µM PCB). Multiple comparisons were adjusted using post hoc Dunnett’s test.

Adjusted R^2^ values, F-statistics, and *p* values are reported for both in vivo and in vitro models. A significance level of α = 0.05 was applied for all statistical analyses, and adjusted *p* values were reported for multiple comparisons.

## Results

### Clinical study

Ten adult subjects with prior occupational exposure to PCBs from the German HELPcB cohort (HELPcB = Health Effects in High-Level Exposure to PCB) and ten adult control subjects with only environmental background exposure to PCBs were enrolled in a single-center clinical study. Demographic data are shown in Supplementary Table 1. The primary objective was to investigate whether systemic exposure to PCBs interacts with CYP-mediated drug metabolism in humans using a CYP activity phenotyping cocktail approach.

Single oral doses of 50 mg caffeine for individual activity assessment of CYP1A2, 50 mg efavirenz for CYP2B6, 2.5 mg torsemide for CYP2C9, 10 mg omeprazole for CYP2C19, 12.5 mg metoprolol for CYP2D6, and 1 mg midazolam for CYP3A were administered simultaneously to the subjects. In addition, a pharmacogenetic analysis was conducted for the genes *CYP1A2*, *CYP2B6*, *CYP2C9*, *CYP2C19*, *CYP2D6*, *CYP3A4*, and *CYP3A* in all subjects. The genotyping results were translated into metabolizer phenotypes, including poor metabolizers (PM), intermediate metabolizers (IM), normal metabolizers (NM), rapid metabolizers (RM), and ultra-rapid metabolizers (UM) for CYP2B6, CYP2C9, CYP2C19, CYP2D6, and CYP3A4 (Supplementary Table 3).

**Fig. 1:**
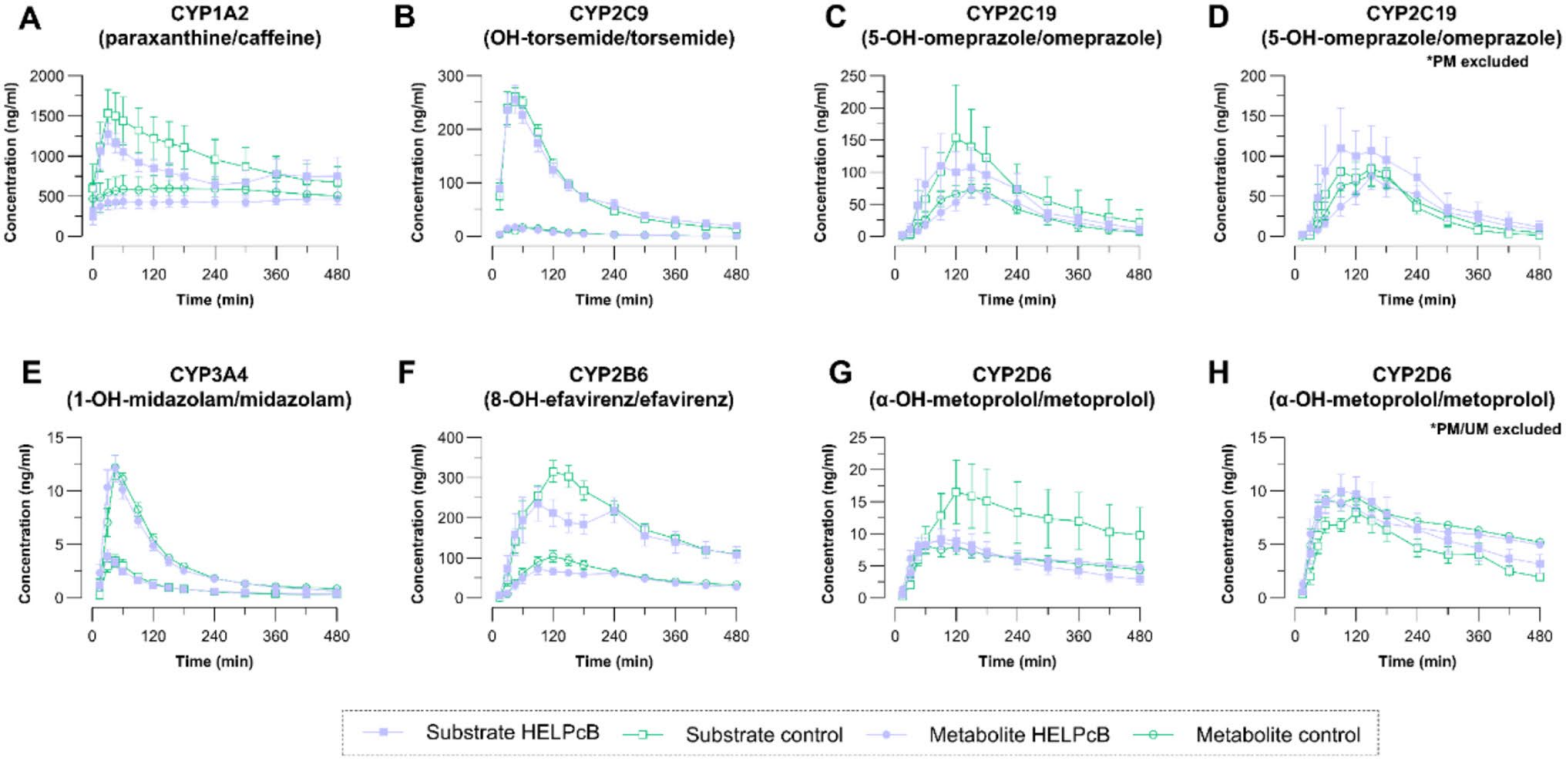
Assessment of cytochrome P450 enzyme (CYP) activity in study participants. Plasma concentration–time profiles for the substrates (squares) and their respective CYP specific metabolites (circles) over a time course of 8 h are shown. Samples of the control group are depicted in green and samples from the HELPcB-group are shown in purple. **A** Caffeine and paraxanthine (CYP1A2), **B** torsemide and hydroxytorsemide (CYP2C9), **C** omeprazole and 5-hydroxyomeprazole (CYP2C19), **D** omeprazole and 5-hydroxyomeprazole (poor metabolizer excluded), **E** midazolam and 1-hydroxymidazolam (CYP3A4), **F** efavirenz and 8-hydroxyefavirenz (CYP2B6), **G** metoprolol and α-hydroxymetoprolol (CYP2D6), **H** metoprolol and α-hydroxymetoprolol (poor and ultra-rapid metabolizer excluded). Concentration of substrates and respective metabolites are given in ng/mL and were obtained after deglucuronidation of the samples. Data are shown as mean ± SEM

Plasma concentrations of the compounds and their respective CYP-mediated metabolites were determined in consecutively taken blood samples up to 24 h after drug administration. Figure 1 presents the plasma concentration–time profiles of the substrates (squares) and their primary CYP-metabolized metabolites (circles) over an 8-h period. The observed profiles followed the expected pattern, with the plasma concentrations of 5-OH-omeprazole/omeprazole (CYP2C19, Fig. 1C) and α-OH-metoprolol/metoprolol (CYP2D6, Fig. 1G) displaying higher variability, mainly related to the different metabolizer phenotypes in these two enzymes. After excluding subjects with genetically determined poor or ultra-rapid metabolizer status, the variabilities in plasma concentrations were substantially smaller (Fig. 1D, H).

Metabolic ratios (MR) were calculated as activity markers for the respective CYP enzyme activities based on the areas under the concentration–time curves between 0 and 8 h (AUC₀₋₈h) as follows: MR AUC₀₋₈h paraxanthine/caffeine for CYP1A2, MR AUC₀₋₈h 8-OH-efavirenz/efavirenz for CYP2B6, MR AUC₀₋₈h OH-torsemide/torsemide for CYP2C9, MR AUC₀₋₈h 5-OH-omeprazole/omeprazole for CYP2C19, MR AUC₀₋₈h α-OH-metoprolol/metoprolol for CYP2D6, and MR AUC₀₋₈h 1-OH-midazolam/midazolam for CYP3A (Supplementary Table 1).

### Plasma concentrations of PCB congeners

**Fig. 2:**
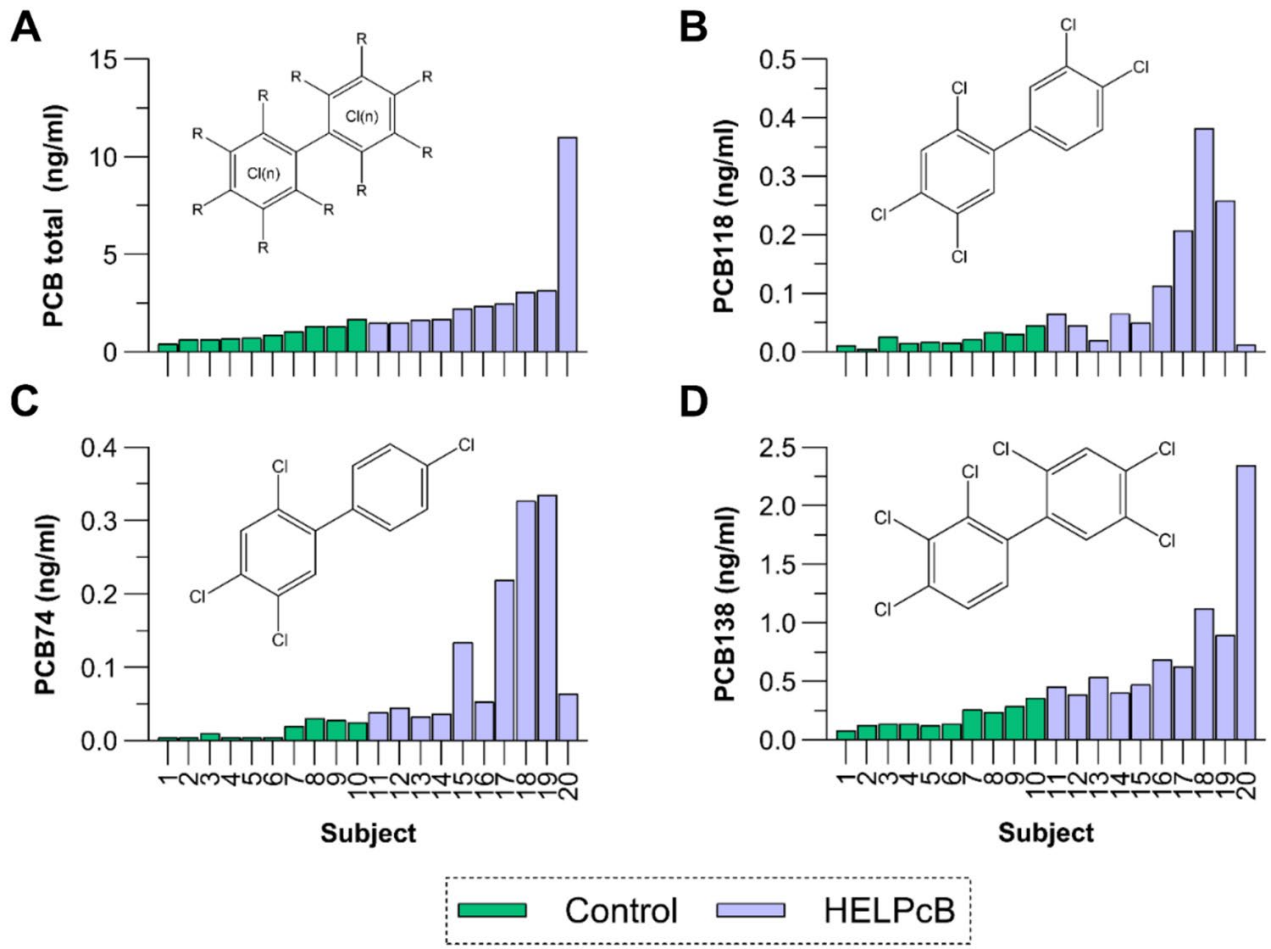
PCB plasma concentrations among study participants. The total PCB levels in study participants and concentrations of PCB74 (lower chlorinated), PCB 118 (dioxin-like) and PCB138 (higher chlorinated) are depicted. PBCs were measured during the recruitment phase of the clinical study. Blue bars indicate former members of the HELPcB group, while green bars represent a control group, reflecting background PCB burden of the general population. PCB concentrations are expressed in ng/ml blood plasma. Regression analyses were conducted to examine the association between cytochrome P450 (CYP) enzyme activities and plasma concentrations of polychlorinated biphenyl (PCB) congeners, including PCB74, PCB118, PCB138, and total PCB. In these analyses, metabolic ratios (MRs) representing CYP activities were treated as dependent variables, while PCB plasma concentrations served as predictor variables (independent variables). The table displays results for separate analyses, including and excluding data for poor metabolizers (PMs) and ultra-rapid metabolizers (UMs) for the respective CYP enzymes. CYP—cytochrome P450, MR—metabolic ratio, AUC_0-8 h_—area under the concentration–time curve from 0 to 8 h, PM—poor metabolizer, UM—ultra-rapid metabolizer, PCB—polychlorinated biphenyl, r^2^ (corrected r^2^)—adjusted coefficient of determination, *t* value (test power) and β (beta, standardized regression coefficient for particular PCB) from regression analysis, *p* value—statistical significance of the regression model

Plasma concentrations of PCB congeners were quantified in blood samples from all participants using gas chromatography/mass spectrometry (GC–MS) (Fig. 2A and Supplementary Table 2). A total of 19 PCB congeners were measured in control (n = 10) and HELPcB (n = 10) participants, with PCBs detected in all individuals. Total PCB plasma concentrations were significantly higher in HELPcB participants (geometric mean = 2.482 ng/mL, range: 1.490–11.007 ng/mL) compared to the control group (geometric mean = 0.865 ng/mL, range: 0.423–1.669 ng/mL) (Supplementary Table 2, Fig. 2A). Notably, some HELPcB individuals exhibited extreme values exceeding 10 ng/mL, highlighting increased PCB exposure in this cohort. In the control group, PCB plasma concentrations primarily reflected background environmental exposure, such as dietary intake, representing the general population’s burden. In contrast, HELPcB participants displayed markedly higher levels of specific PCB congeners, particularly PCB118 (Fig. 1B), PCB74 (Fig. 1C), and PCB138 (Fig. 1D), all of which were consistently detected above the lower limit of quantification (LLOQ) in all participants and were significantly elevated in HELPcB individuals compared to controls (Table 1, Supplementary Material).

**Table 1:** Associations between cytochrome P450 (CYP) enzyme activities and plasma polychlorinated biphenyl (PCB) concentrations

CYP activity	Metabolic ratio AUC_0–8 h_	PCB total			PCB74			PCB118			PCB138		
		Corrected r^2^	*t* value/β	*p* value	Corrected r^2^	*t* value/β	*p* value	Corrected r^2^	*t* value/β	*p* value	Corrected r^2^	*t* value/β	*p* value
CYP1A2	Paraxanthine/caffeine	− 0.043	0.458/0.108	0.653	0.062	− 1.502/− 0,334	0.150	0.155	− 2.115/− 0,446	0.049	− 0.053	0,221/0,052	0.827
CYP2B6	8-OH-efavirenz/efavirenz	0.029	− 1,215/− 0,299	0.243	− 0.022	0,808/0,204	0.432	0.003	1,020/0,255	0.324	0.022	− 1,164/− 0,288	0.262
	8-OH-efavirenz/efavirenz PM excl	0.064	− 1,140/− 0,362	0.185	− 0.046	0,622/0,170	0.545	− 0.022	0,839/0,227	0.417	0.063	− 1,395/− 0,361	0.186
CYP2C9	OH-torsemide/torsemide	− 0.046	0,404/0,095	0.691	0.109	1,826/0,395	0.085	0.040	1,339/0,301	0.197	− 0.049	0,341/0,080	0.737
	OH-torsemide/torsemide PM excl	− 0.053	0,314/0,076	0.757	0.099	1,723/0,386	0.103	0.027	1,228/0,286	0.236	− 0.056	0,219/0,053	0.830
CYP2C19	5-OH-omeprazol/omeprazol	− 0.053	− 0,224/− 0,053	0.826	− 0.030	− 0,666 − 0,155	0.514	− 0.023	− 0,757/− 0,176	0.459	− 0.050	− 0,300/− 0,071	0.768
	5-OH-omeprazol/omeprazol PM excl	− 0.048	− 0,417/− 0,101	0.682	− 0.009	− 0,916/− 0,217	0.372	− 0.002	− 0,980/− 0,231	0.341	− 0.040	− 0,550/− 0,132	0.589
CYP2D6	α-OH-metoprolol/metoprolol	− 0.050	− 0,302/− 0,071	0.766	0.094	1,723/0,376	0.102	0.163	2,169/0,455	0.044	− 0.054	0,157/0,037	0.877
	α-OH-metoprolol/metoprolol PM/UM excl	0.051	− 1,326/− 0,345	0.208	− 0.074	− 0,200/− 0,055	0.844	− 0.065	− 0,382/− 0,105	0.709	0.067	− 1,418/− 0,366	0.180
CYP3A4	1-OH-midazolam/midazolam	− 0.047	− 0,380/− 0,089	0.709	− 0.014	− 0,858/− 0,198	0.402	0.024	− 1,210/− 0,274	0.242	− 0.034	− 0,614/− 0,143	0.547

### Clinical effects of PCB118 and PCB74 exposure on CYP1A2 and CYP2C9 activities

The clinical effects of PCB exposure on CYP activities, as represented by the respective MRs, were explored using linear regression analysis (Table 1). For the regression analysis, CYP activities were used as dependent variables, while plasma concentrations of PCB congeners PCB74, PCB118, PCB138, and total PCB were treated as independent variables. Regression models were performed both with and without PMs and UMs for the respective CYPs as shown in Table 1.

A statistically significant association was observed between PCB118 plasma concentrations and CYP1A2 activity (R^2^ = 0.155, t = − 2.115, p = 0.049, β = − 0.446), indicating that higher PCB118 levels are associated with a decrease in CYP1A2 activity. This suggests that PCB118 exposure may influence CYP1A2-mediated metabolism, particularly the conversion of caffeine to paraxanthine. Although the proportion of variability explained by PCB118 is moderate (~ 15.5%), this result supports a potential PCB–drug interaction involving substances metabolized by CYP1A2.

In contrast, a weaker association was observed between PCB74 plasma concentrations and CYP2C9 activity (excluding PMs) (R^2^ = 0.099, t = 1.723, p = 0.103, β = 0.386). While this effect was not statistically significant, the direction and magnitude of the β-coefficient suggest a possible positive relationship between PCB74 levels and CYP2C9 activity. Although the proportion of variability explained is relatively small, the relatively high *t* value indicates some effect in the expected direction. The current sample size may not be sufficient to detect a statistically significant association. Further studies with larger cohorts are needed to determine whether this trend reaches statistical significance.

**Fig. 3:**
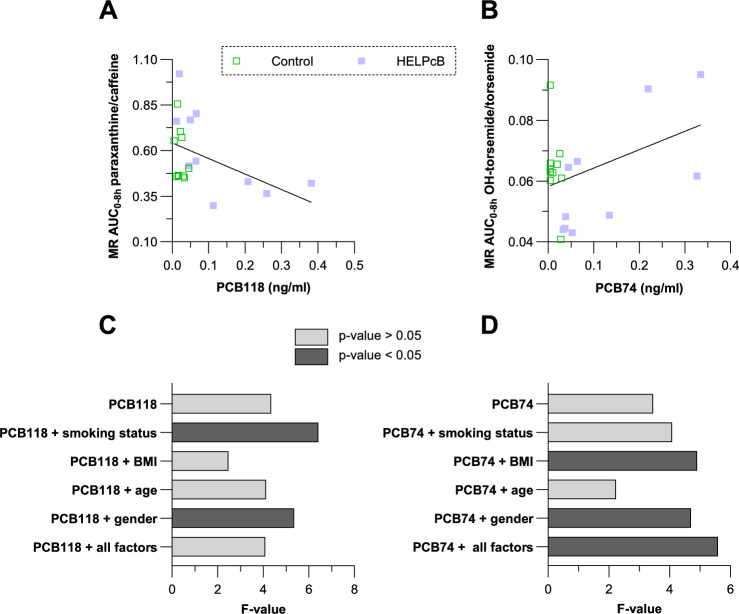
Linear correlation of PCB118 and PCB74 plasma concentrations with metabolic ratios of paraxanthine/caffeine and OH-torsemide/torsemide. The bivariate linear regression analyzes the relationship between plasma concentrations of PCB congeners 118 and 74 (independent variables) and metabolic ratios (MR, a proxy for CYP enzyme activity; dependent variable). Panels A and B present the correlations between PCB118/CYP1A2 and PCB74/CYP2C9, respectively. Green squares represent control subjects, while blue squares indicate HELPcB-exposed individuals. Panels C and D show *F* values from linear mixed models assessing the impact of PCB118 on CYP1A2 (C) and PCB74 on CYP2C9 (D) while adjusting for covariates (smoking status, BMI, age, and gender). Different grayscale shades indicate statistical significance: darker bars represent significant results (*p* < 0.05), while lighter bars indicate non-significant results (*p* > 0.05)

Figure 3 illustrates the decrease in CYP1A2 activity with increasing PCB118 plasma concentrations (Fig. 3A) and a numerical trend toward increased CYP2C9 activity with increasing PCB74 plasma concentrations (Fig. 3B).

To further refine these findings, a hierarchical generalized mixed model (HGMM) was applied to account for repeated measures and inter-individual variability. This model allowed for the assessment of PCB effects while adjusting for potential confounders, including smoking status and gender (categorical variables) as well as BMI and age (continuous variables). Variance analysis within the mixed model framework identified co-factors that potentially influence CYP activities. Darker bars in the forest plots of Fig. 3C, D indicate significant effects (*p* < 0.05), while lighter bars represent non-significant effects (*p* > 0.05).

For PCB118 and CYP1A2, the association was initially non-significant but became significant when smoking status was included as a co-factor, suggesting that smoking plays a key role in modulating the effect of PCB118 on CYP1A2 activity. This association remained significant when gender was included, indicating that both factors contribute to inter-individual variability in CYP1A2 activity.

For PCB74 and CYP2C9, the association was initially non-significant but became significant when BMI, gender, or all factors were included as co-factors. This suggests that metabolic factors such as body composition (reflected by BMI) and sex-specific differences in CYP expression or hormonal influences may contribute to inter-individual variability in CYP2C9 activity following PCB74 exposure.

In summary, this analysis suggests that exposure to PCB118 and PCB74 may lead to distinct activity changes in CYP1A2 and CYP2C9 in humans, potentially resulting in pharmacokinetic interactions with drugs or other compounds metabolized by these enzymes. While PCB118 significantly decreases CYP1A2 activity, which may have implications for drugs metabolized by this enzyme (e.g., caffeine, theophylline and certain antidepressants), PCB74 may enhance CYP2C9 activity.

### Effects of PCB118 and PCB74 on CYP1A2 and CYP2C9 activity in vitro

**Fig. 4:**
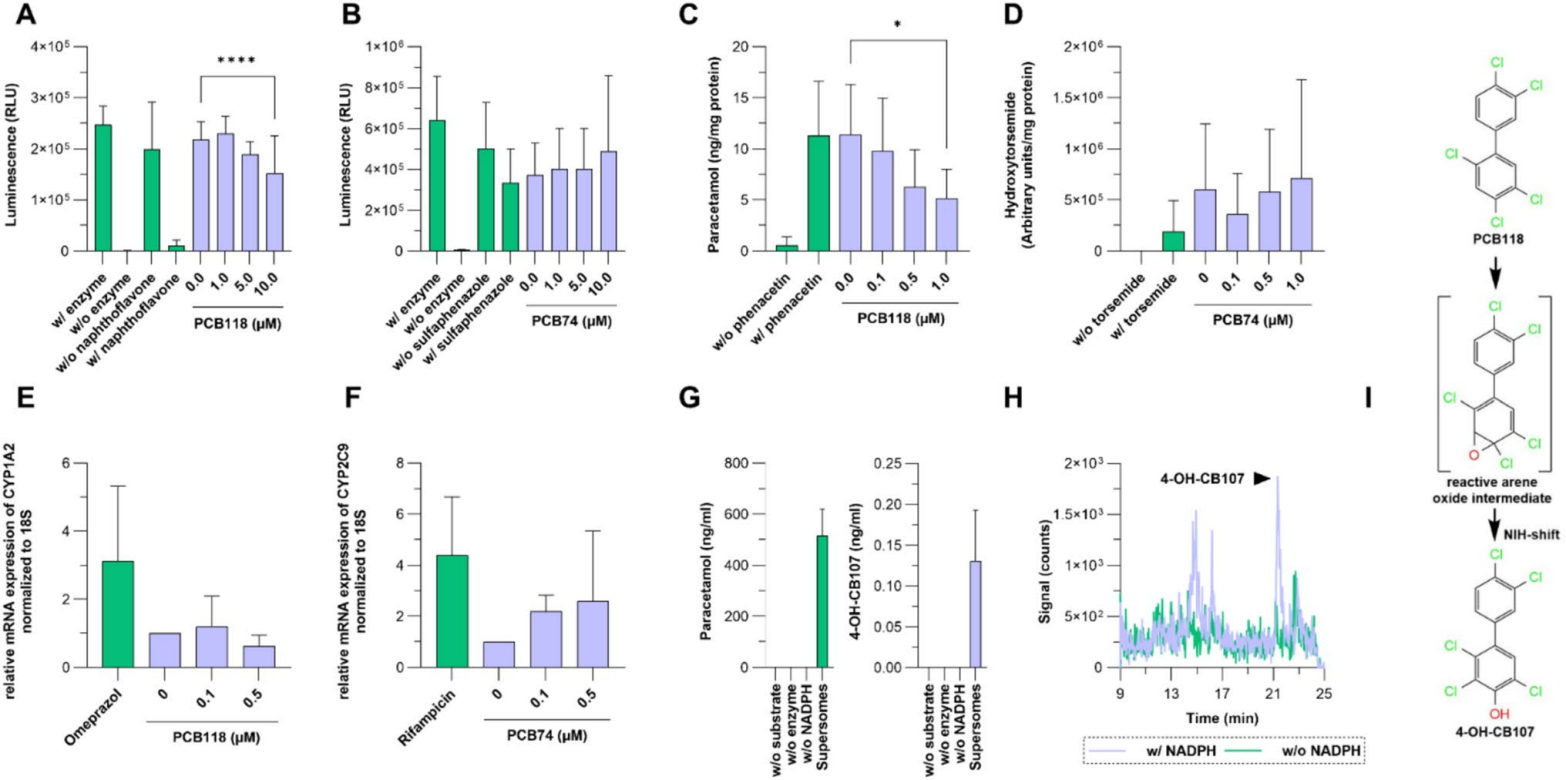
In vitro assessment of PCB74 and PCB118 on CYP1A2 and CYP2C9 activity, expression, and metabolism. The effects of PCB74 and PCB118 on CYP1A2 and CYP2C9 were assessed using luminescence-based enzyme assays, transgenic cell models, and primary human hepatocytes. **A, B** Luciferase-based enzyme assays were conducted with recombinant CYP1A2 (**A**) and CYP2C9 (**B**) using the luminogenic substrate Luciferin-ME. Positive controls included α-naphthoflavone (CYP1A2) and sulfaphenazole (CYP2C9). **C, D** CYP activity was analyzed in transgenic HEK293 cells overexpressing CYP1A2 (**C**) or CYP2C9 (**D**) using phenacetin and torsemide as respective probe substrates. **E, F** mRNA expression of CYP1A2 (**E**) and CYP2C9 (**F**) was measured in primary human hepatocytes treated with PCB74 or PCB118 (0.1–0.5 µM). Omeprazole and rifampicin were included as positive controls for CYP1A2 and CYP2C9 induction, respectively. **G** CYP1A2 enzyme activity was assessed using phenacetin (10 µM) as probe substrate and tested for PCB118 metabolism. **H** CYP1A2-mediated metabolism of PCB118 was analyzed, with the detection of 4-OH-CB107, a hydroxylated metabolite formed via an NIH 1,2-shift mechanism. NADPH dependency was tested by omitting NADPH in control incubations. I Proposed metabolic pathway illustrates the CYP1A2-mediated hydroxylation of PCB118, leading to the formation of 4-OH-CB107 via a reactive arene oxide intermediate. All data shown with mean [SD]

To assess the potential of PCB74 and PCB118 to modulate CYP1A2 and CYP2C9 activity in vitro, we first performed luminescence-based enzyme assays using recombinant CYPs and the specific luminogenic substrate Luciferin–ME. As shown in Fig. 4A, PCB118 (1–10 µM) exhibited a concentration-dependent inhibitory effect on CYP1A2 enzyme activity, leading to a significant reduction at the highest concentration tested (p < 0.0001). In contrast, CYP2C9 activity remained unaffected by PCB74 exposure (1–10 µM), with activity levels showing no significant deviation from control conditions (Fig. 4B). The inclusion of known CYP inhibitors (α-naphthoflavone for CYP1A2, sulfaphenazole for CYP2C9) as positive controls confirmed the assay validity.

To further investigate these effects in a physiologically relevant system, we utilized transgenic HEK293 cell lines overexpressing either CYP1A2 or CYP2C9. CYP1A2 activity was assessed using phenacetin as a substrate, while CYP2C9 activity was validated using torsemide. As shown in Fig. 4C, PCB118 significantly inhibited CYP1A2 activity in a dose-dependent manner, with 1.0 µM PCB118 reducing enzyme activity (p < 0.05, p < 0.001). In contrast, PCB74 did not significantly modulate CYP2C9 activity in transgenic cells, as depicted in Fig. 4D.

In addition to enzymatic activity, we evaluated whether PCB74 and PCB118 modulate CYP expression at the transcriptional level in primary human hepatocytes derived from three independent donor isolates. As shown in Fig. 4F, PCB74 treatment (0.1 and 0.5 µM) did not significantly increase CYP2C9 mRNA expression, although a trend toward upregulation was observed. This suggests that PCB74 may induce CYP2C9 expression, though high variability among donor samples may have limited statistical significance.

Similarly, we assessed the effect of PCB118 on CYP1A2 mRNA expression in primary human hepatocytes. As shown in Fig. 4E, PCB118 treatment (0.1–0.5 µM) did not significantly alter CYP1A2 mRNA levels, suggesting no effect on the basal expression of CYP1A2 in hepatocytes. In contrast, omeprazole, a well-established CYP1A2 inducer, strongly upregulated CYP1A2 expression, confirming the expected responsiveness of the hepatocyte system. These findings indicate that PCB118 does not modulate CYP1A2 gene expression at the transcriptional level, further supporting the notion that its inhibitory effect is exerted primarily at the enzymatic level rather than through transcriptional regulation.

Taken together, our findings indicate that PCB118 significantly inhibits CYP1A2 activity in both luminescence-based assays and transgenic cell models, while PCB74 does not significantly affect CYP2C9 activity but may show a trend toward upregulating its expression in primary human hepatocytes. Furthermore, PCB118 does not influence the basal expression of CYP1A2, reinforcing the conclusion that its inhibitory effects are mediated at the post-transcriptional or enzymatic level rather than through gene regulation.

To determine whether PCB118 serves as a substrate for CYP1A2 and could act as a competitive inhibitor for other CYP1A2 substrates, we conducted in vitro metabolism experiments. Human CYP1A2, coexpressed with P450 reductase in insect cells (Supersomes™), was incubated with phenacetin or caffeine for 1 h, PCB28 as a reference substrate (data not shown), or PCB118 for 1 and 4 h, and the supernatants were collected for analysis. As expected, CYP1A2 efficiently catalyzed the O-de-ethylation of phenacetin to paracetamol, confirming enzymatic activity (Fig. 4G). In parallel, CYP1A2-mediated metabolism of PCB118 was assessed. As shown in Fig. 4H, CYP1A2 catalyzed the hydroxylation of PCB118, leading to the formation of 4-OH-CB107, a hydroxylated product generated via an NIH 1,2-shift. The formation of 4-OH-CB107 was strictly NADPH-dependent, as demonstrated by the absence of this metabolite in control incubations lacking NADPH, substrate, or enzyme. The identity of 4-OH-CB107 was verified by comparison with an authentic reference standard, showing identical retention time and spectral characteristics (supplemental Fig. 1).

The proposed metabolic conversion of PCB118 to 4-OH-CB107 via a reactive arene oxide intermediate is depicted in Fig. 4I. The demonstration that PCB118 is a substrate of CYP1A2 provides a mechanistic explanation for the previously observed in vitro and in vivo inhibition results. Since PCB118 undergoes CYP1A2-mediated metabolism, it is likely that PCB118 competes with other CYP1A2 substrates, such as phenacetin and caffeine, at the enzymes active site, thereby acting as a competitive inhibitor of CYP1A2. This hypothesis is supported by the observed reduction in CYP1A2-dependent probe substrate metabolism upon PCB118 exposure, as demonstrated in luminescence-based assays and transgenic cell models (Fig. 4A, C).

## Discussion

This study demonstrates that PCB118 and PCB74 influence CYP-mediated drug metabolism, with PCB118 acting as a significant inhibitor of CYP1A2, and that PCB74 may affect CYP2C9-mediated drug metabolism by potentially modulating its expression. These findings highlight the capacity of persistent organic pollutants (POPs) to interfere with drug metabolism, with possible implications for therapeutic efficacy and patient safety.

The pharmacokinetic study revealed that plasma concentrations of PCB118 showed a linear relationship to CYP1A2 activity, as measured by caffeine metabolism. Higher PCB118 plasma concentrations were associated with reduced CYP1A2 activity in the linear correlation, but no statistically significant differences were detected in the grouped comparison between the control and HELPcB groups. It should be noted that basal caffeine concentrations differed between the groups, most likely reflecting variable baseline caffeine intake and incomplete adherence to the caffeine–abstinence protocol on the study day. This may have contributed to the observed differences in Cmax values and the slight surge in caffeine and paraxanthine levels at 6 h in the HELPcB group. However, because CYP1A2 activity was assessed using AUC_0–8 h_ metabolic ratios, these baseline variations are largely compensated by corresponding increases in metabolite formation.

In vitro assays confirmed the effect of PCB118, showing a dose-dependent inhibition of CYP1A2 activity. These results align with previous reports indicating that dioxin-like PCBs, including PCB118 can inhibit CYP1A2 activity in vitro, suggesting that PCB118 may compete with substrates by directly interfering with enzymatic binding sites (Staskal et al. 2005). Supporting this notion a similar but weaker association (p = 0.068) between reduced CYP1A2 activity and increased PCB118 plasma concentrations was previously observed in a Faroese Islands cohort using caffeine-based CYP1A2 phenotyping; however, despite PCB levels being approximately tenfold higher in that cohort, the association was less pronounced than in the present study (Petersen et al. 2006). Possible explanations include differences in exposure duration, co-exposures, genetic variability in CYP1A2 activity, or nonlinear dose–response relationships. Further studies are required to clarify these discrepancies and determine whether PCB118 exhibits threshold-dependent effects on CYP1A2 inhibition.

An important aspect when interpreting the in vitro*–*in vivo relationship is the potential for hepatic sequestration of PCB118. Dioxin-like PCB congeners are known to accumulate in the liver, and previous work by Diliberto et al. demonstrated in a CYP1A2 knock-out mouse model that high-affinity binding of dioxin-like polyhalogenated aromatic hydrocarbons (including TCDD) to CYP1A2 mediates their hepatic retention (Diliberto et al. 1997). In the absence of CYP1A2, no hepatic accumulation was observed, and the compounds were redistributed predominantly to adipose tissue. Although this mechanism has not been directly demonstrated for PCB118, the fact that our data identify PCB118 as a CYP1A2 substrate makes it plausible that a similar process may occur. If so, the hepatic concentration of PCB118 in vivo could be substantially higher than plasma levels, which may at least partially explain the higher concentrations required to inhibit CYP1A2 in isolated systems compared to primary human hepatocytes and the observed discrepancy with plasma concentrations in exposed individuals.

In addition to hepatic sequestration, a mechanism-based inhibition (MBI) may provide a plausible explanation for the discrepancy between the in vitro and in vivo findings. Structural studies have shown that highly chlorinated, planar dioxins such as TCDD can bind with high affinity to CYP1A2 and induce conformational changes that close the enzyme’s access and exit channels, effectively blocking substrate turnover (Swigonska et al. 2022). Since PCB118 is structurally dioxin-like and our experiments demonstrated that it binds as a substrate to CYP1A2 and forms a reactive arene oxide, it is conceivable that a similar process occurs here. PCB118 may occupy the active site, promote channel closure and thereby cause a quasi-irreversible inactivation of CYP1A2. Such a mechanism could explain why even relatively low in vivo PCB118 concentrations are associated with stronger inhibition than predicted from conventional in vitro data.

This interpretation is further supported by the finding that PCB118 undergoes metabolism via an NIH-shift mechanism. This transformation involves an initial oxidation step, leading to the formation of an arene oxide intermediate, which subsequently rearranges to 4-OH-CB107, a hydroxylated metabolite (Haraguchi et al. 1998). The formation of 4-OH-CB107 was strictly NADPH-dependent, confirming that PCB118 undergoes CYP1A2-mediated hydroxylation. Given the high reactivity of arene oxides, it is plausible that these intermediates may form covalent bonds with the catalytic center of CYP enzymes, potentially leading to irreversible enzyme inactivation—a characteristic often associated with mechanism-based inhibition (Guengerich 2001; Kalgutkar et al. 2005; Deodhar et al. 2020). This suggests that PCB118 not only competes with other CYP1A2 substrates but may also contribute to long-term suppression of CYP1A2 activity, necessitating de novo enzyme synthesis for recovery (Villa-Zapata et al. 2022).

The pharmacological relevance of CYP1A2 inhibition is well-documented. A pharmacovigilance study by Villa-Zapata et al. (2022) found that co-administration of tizanidine with strong CYP1A2 inhibitors, such as ciprofloxacin or fluvoxamine, resulted in severe pharmacodynamic effects, including hypotension and bradycardia. This underscores the clinical significance of CYP1A2 inhibition, as reduced enzyme activity can dramatically increase plasma drug concentrations, leading to enhanced drug effects and toxicity. In the context of this study, PCB118-induced CYP1A2 inhibition may similarly lead to increased systemic exposure to CYP1A2-metabolized drugs, with potential consequences for drug efficacy and safety.

The role of genetic polymorphisms in CYP1A2 activity further reinforces the complexity of interindividual variability in PCB–drug interactions. A study by Yang et al. (2024) investigated the influence of CYP1A2 polymorphisms on pirfenidone metabolism and found that individuals carrying the CYP1A2*1F AA genotype exhibited increased CYP1A2 activity, resulting in lower pirfenidone plasma concentrations and fewer adverse drug reactions (ADRs). This suggests that genetic variation may modify PCB118’s impact on CYP1A2 inhibition, making individuals with inherently low CYP1A2 activity more vulnerable to PCB-induced pharmacokinetic disruptions.

In contrast to the effect of PCB118 on CYP1A2, PCB74 did not significantly affect CYP2C9 activity, although a trend toward increased CYP2C9 mRNA expression was observed in primary human hepatocytes. While this effect was not statistically significant, it suggests that PCB74 may act at the transcriptional level rather than directly inhibiting or inducing enzymatic activity. The regulation of CYP2C9 expression can be influenced by various factors, including electrophilic stress and activation of transcription factors such as activator protein 1 (AP-1). Studies have shown that electrophilic xenobiotics can induce CYP2C9 expression through AP-1 activation, involving proteins, such as cFos and JunD (Makia et al. 2014). For instance, Makia et al. demonstrated that the electrophilic compound tert-butylhydroquinone (tBHQ) induces CYP2C9 expression via AP-1 activation, with cFos and JunD binding to specific sites on the CYP2C9 promoter. Although the exact mechanism by which PCB74 may modulate CYP2C9 expression remains to be elucidated, these findings highlight the potential for environmental contaminants to alter drug metabolism by affecting gene transcription pathways (Makia et al. 2014). Given that other environmental contaminants, such as dioxins, upregulate CYP expression over time, future studies should investigate whether longer exposure durations or higher PCB74 concentrations might enhance this effect.

While environmental toxicants have traditionally been studied for their chronic effects, this study demonstrates that PCBs can also exert acute pharmacokinetic effects, particularly when interacting with co-administered drugs (Villa-Zapata et al. 2022). Although PCBs accumulate in human tissues over decades, their ability to modulate CYP enzyme activity in a dose-dependent manner suggests a dynamic interplay between chronic exposure and acute metabolic disruptions.

Notably, the hierarchical generalized mixed model analysis also provided insights into the role of covariates, such as smoking status, BMI and gender in modulating susceptibility to PCB effects (Fig. 3). Inclusion of smoking status as a covariate increased the *F* value for PCB118 in relation to CYP1A2 activity, suggesting that smokers may be more susceptible to PCB118-mediated CYP1A2 inhibition. In contrast, inclusion of BMI reduced the *F* value, indicating that higher BMI attenuates this effect. Gender also contributed to variability, with female sex slightly enhancing the PCB118 effect. For PCB74 and CYP2C9, BMI and gender together increased the model fit, suggesting that body composition and sex-specific CYP2C9 expression may modulate susceptibility to PCB74.

This raises broader concerns about whether similar mechanisms apply to other widespread environmental contaminants. Many POPs have been shown to disrupt drug metabolism by altering CYP activity, either through direct inhibition or modulation of gene expression. Among these, per- and polyfluoroalkyl substances (PFAS) have gained increasing attention due to their global environmental contamination and potential toxicological effects. Although PCBs and PFAS differ structurally—PCBs being chlorinated biphenyls and PFAS consisting of fluorinated alkyl chains—both classes of compounds share key properties, including high environmental persistence, bioaccumulation, and the potential to interfere with biological detoxification systems.

PFAS have been shown to interact with CYP enzymes through both inhibition and induction (Amstutz et al. 2022; Hvizdak et al. 2023; Solan and Lavado 2023). For instance, PFOS inhibits CYP1A2 activity, similar to PCB118, while certain PFAS variants induce CYP3A4 expression (Solan and Lavado 2023). However, the mechanisms by which PCBs and PFAS modulate CYP activity may differ. While PCB118 inhibition appears to be competitive or mechanism-based, some PFAS compounds may alter CYP function through conformational changes or activation of the aryl hydrocarbon receptor (AhR). Given the widespread and often overlapping contamination with PCBs and PFAS, future research should investigate potential additive or synergistic effects on CYP-mediated metabolism, particularly in chronically exposed populations.

This study provides evidence that PCB118 significantly inhibits CYP1A2 activity, while PCB74 may weakly induce CYP2C9 expression. Furthermore, the metabolism of PCB118 via an NIH shift suggests a possible mechanism-based inhibition of CYP1A2, which could have long-lasting pharmacokinetic consequences. These findings underscore the importance of considering environmental contaminant exposure in pharmacotherapy, as such exposures can influence drug efficacy and safety by modulating metabolic enzyme activity. Future pharmacokinetic modeling should account for both chronic accumulation and acute metabolic perturbations, particularly for drugs with a narrow therapeutic index that rely on CYP1A2 or CYP2C9 for clearance. Given that many environmental contaminants co-exist in human tissues, future research should investigate how multiple xenobiotics may collectively modulate CYP enzyme activity, potentially compounding pharmacokinetic variability and drug safety concerns.

## Supplementary Information

Below is the link to the electronic supplementary material.

Supplementary file1 (DOCX 414 KB)
