# Supplementary material for: Polychlorinated biphenyls (PCBs) interact with drug metabolism in vivo
Source: Arch Toxicol. 2025 Aug 15;99(11):4423–37. doi: 10.1007/s00204-025-04145-6 (PMC12477080; doi:10.1007/s00204-025-04145-6)
Supplement: Supplementary file 1 — Supplementary file1 (DOCX 414 KB) [file 204_2025_4145_MOESM1_ESM.docx]

**Supplemental Information**

**Polychlorinated biphenyls (PCBs) interact with drug metabolism *in vivo.***

*Julian Peter Müller ^1,a^, Jens Rengelshausen^1,b^, Salah Laieb^1,b^, Maryam Safavi^b^, Andrea Kaifie^b^, Andre Esser^b^, Thomas Schettgen^b^, Jens Bertram^b^, Julia Krabbe^b^, Elke Schaeffeler^c^, Jens Sarömb^a^, Katja S. Just^a^, Roman Tremmel^c^, Matthias Schwab^c,d^, Julia C. Stingl^a,e^, Thomas Kraus^b^ and Patrick Ziegler^b^*

^a^Institute of Clinical Pharmacology, University Hospital of RWTH, 52074, Aachen, Germany

^b^Institute for Occupational, Social and Environmental Medicine, Medical Faculty, RWTH Aachen University, Pauwelsstrasse 30, 52074, Aachen, Germany

^c^Dr. Margarete Fischer-Bosch-Institute of Clinical Pharmacology, Stuttgart and University of Tuebingen, Tuebingen, Germany

^d^Departments of Clinical Pharmacology, and Pharmacy and Biochemistry, University of Tuebingen, Tuebingen, Germany

^e^ Present address: Department of Clinical Pharmacology and Pharmacoepidemiology, University Hospital Heidelberg, Heidelberg, Germany

^+^ authors contributed equally

Correspondence to: Patrick Ziegler, Institute for Occupational, Social and Environmental Medicine, Medical Faculty, RWTH Aachen University, Pauwelsstrasse 30, 52074, Aachen, Germany

**Email:** [pziegler@ukaachen.de](mailto:pziegler@ukaachen.de)

Supplemental table 1: Study demographics of control and HELPcB cohort. Metabolic ratios of metabolite to parent drug and the PCB exposure are shown.

| **Parameter** | **Mean [±SD] all** | **N all** | **Mean [±SD] HELPcB** | **N HELPcB** | **Mean [±SD] control** | **N control** | **p-value (U-test)** |
| --- | --- | --- | --- | --- | --- | --- | --- |
| Age (years) | 57.95 ± 9.01 | 20 | 59.60 ± 11.45 | 10 | 56.30 ± 5.85 | 10 | 0.190 |
| Weight (kg) | 83.15 ± 16.38 | 20 | 89.70 ± 18.81 | 10 | 76.60 ± 10.84 | 10 | 0.105 |
| BMI (kg/m^2^) | 27.30 ± 4.15 | 20 | 29.08 ± 4.64 | 10 | 25.53 ± 2.80 | 10 | 0.089 |
| (CYP1A2) MR AUC_0-8h_ paraxanthine/caffeine | 0.58 ± 0.19 | 20 | 0.59 ± 0.23 | 10 | 0.57 ± 0.14 | 10 | 1.000 |
| (CYP2B6) MR AUC_0-8h_ 8-OH-efavirenz/efavirenz | 0.30 ± 0.10 | 17 | 0.29 ± 0.08 | 10 | 0.30 ± 0.11 | 10 | 1.000 |
| (CYP2B6) MR AUC_0-8h_ 8-OH-efavirenz/efavirenz PM excl. | 0.31 ± 0.10 | 15 | 0.30 ± 0.08 | 6 | 0.32 ± 0.11 | 9 | 1.000 |
| (CYP2C9) MR AUC_0-8h_ OH-torsemide/torsemide | 0.06 ± 0.02 | 20 | 0.06 ± 0.02 | 10 | 0.06 ± 0.01 | 10 | 0.529 |
| (CYP2C9) MR AUC_0-8h_ OH-torsemide/torsemide PM excl. | 0.06 ± 0.02 | 19 | 0.06 ± 0.02 | 10 | 0.07 ± 0.01 | 9 | 0.278 |
| (CYP2C19) MR AUC_0-8h_ 5-OH-omeprazol/omeprazol | 1.08 ± 0.74 | 20 | 0.94 ± 0.44 | 10 | 1.23 ± 0.96 | 10 | 0.579 |
| (CYP2C19) MR AUC_0-8h_ 5-OH-omeprazol/omeprazol PM excl. | 1.14 ± 0.72 | 19 | 0.94 ± 0.44 | 10 | 1.36 ± 0.92 | 9 | 0.315 |
| (CYP2D6) MR AUC_0-8h_ α-OH-metoprolol/metoprolol | 1.57 ± 1.28 | 20 | 1.78 ± 1.39 | 10 | 1.35 ± 1.19 | 10 | 0.529 |
| (CYP2D6) MR AUC_0-8h_ α-OH-metoprolol/metoprolol PM/UM excl. | 1.53 ± 0.80 | 15 | 1.43 ± 0.89 | 9 | 1.68 ± 0.69 | 6 | 0.607 |
| (CYP3A4) MR AUC_0-8h_ 1-OH-midazolam/midazolam | 4.22 ± 2.54 | 20 | 4.04 ± 2.79 | 10 | 4.41 ± 2.40 | 10 | 0.529 |
| PCB sum (ng/ml) | 2.00 ± 2.26 | 20 | 3.07 ± 2.86 | 10 | 0.936 ± 0.392 | 10 | 0.001 |
| PCB74 (ng/ml) | 0.071 ± 0.102 | 20 | 0.129 ± 0.121 | 10 | 0.014 ± 0.011 | 10 | 0.001 |
| PCB118 (ng/ml) | 0.072 ± 0.099 | 20 | 0.122 ± 0.122 | 10 | 0.022 ± 0.012 | 10 | 0.005 |
| PCB138 (ng/ml) | 0.493 ± 0.516 | 20 | 0.795 ± 0.593 | 10 | 0.191 ± 0.090 | 10 | 0.001 |

Mann-Whitney U-test was used to assess statistical significance between control and HELPcB group. N: number of participants, MR: metabolic ratio, AUC_0-8h_: area under the curve from 0 to 8 hours, PM: poor metabolizer, UM: ultratrapid metabolizer

Supplemental table 2: Detailed depiction of PCB plasma concentrations of the study participants sorted into control and HELPcB group.

| **Control** | | | | | | **HELPcB** | | | | |
| --- | --- | --- | --- | --- | --- | --- | --- | --- | --- | --- |
| Congener | N | Geometric mean | IQR (25-75%) | Total range (min - max) | | N | Geometric mean | IQR (25-75%) | Total range (min - max) | |
| PCB 28 | 10 | 0.006 | 0.000 | 0.005 | 0.014 | 10 | 0.014 | 0.051 | 0.005 | 0.251 |
| PCB 52 | 10 | BLQ | | | | 10 | BLQ | | | |
| PCB 101 | 10 | BLQ | | | | 10 | 0.005 | 0.000 | 0.005 | 0.010 |
| PCB 153 | 10 | 0.297 | 0.237 | 0.140 | 0.668 | 10 | 0.919 | 0.425 | 0.592 | 3.769 |
| PCB 138 | 10 | 0.173 | 0.123 | 0.086 | 0.361 | 10 | 0.672 | 0.382 | 0.388 | 2.346 |
| PCB 180 | 10 | 0.372 | 0.229 | 0.183 | 0.622 | 10 | 0.808 | 0.358 | 0.405 | 4.877 |
| PCB 81 | 10 | BLQ | | | | 10 | BLQ | | | |
| PCB 77 | 10 | BLQ | | | | 10 | BLQ | | | |
| PCB 123 | 10 | BLQ | | | | 10 | BLQ | | | |
| PCB 118 | 10 | 0.019 | 0.014 | 0.005 | 0.045 | 10 | 0.075 | 0.138 | 0.013 | 0.382 |
| PCB 114 | 10 | BLQ | | | | 10 | 0.008 | 0.010 | 0.005 | 0.019 |
| PCB 105 | 10 | BLQ | | | | 10 | 0.014 | 0.040 | 0.005 | 0.101 |
| PCB 126 | 10 | BLQ | | | | 10 | BLQ | | | |
| PCB 167 | 10 | 0.008 | 0.006 | 0.005 | 0.021 | 10 | 0.027 | 0.013 | 0.014 | 0.054 |
| PCB 156 | 10 | 0.036 | 0.028 | 0.021 | 0.072 | 10 | 0.097 | 0.048 | 0.052 | 0.415 |
| PCB 157 | 10 | 0.005 | 0.000 | 0.005 | 0.010 | 10 | 0.016 | 0.009 | 0.005 | 0.069 |
| PCB 169 | 10 | BLQ | | | | 10 | BLQ | | | |
| PCB 189 | 10 | 0.007 | 0.006 | 0.005 | 0.013 | 10 | 0.014 | 0.008 | 0.005 | 0.078 |
| PCB 74 | 10 | 0.010 | 0.019 | 0.005 | 0.030 | 10 | 0.086 | 0.158 | 0.033 | 0.335 |
| PCB Total | 10 | 0.865 | 0.592 | 0.423 | 1.669 | 10 | 2.482 | 1.258 | 1.490 | 11.007 |

All values are given in ng/ml with the geometric mean, interquartile range (IQR) and total range depicted. The respective PCB congener is assigned as BLQ if it was detected in none of the participants from the respective group (control, HELPcB) N: number of participants, IQR: interquartile range, min: minimum, max: maximum, BLQ: below limit of quantification

| **Subject** | **Group** | **CYP1A2 diplotype** | **CYP2A6 diplotype** | **CYP2B6 diplotype** | **CYP2B6 predicted phenotype** | **CYP2C9 diplotype** | **CYP2C9 predicted phenotype** | **CYP2C19 diplotype** | **CYP2C19 activity score** | **CYP2C19 predicted phenotype** | **CYP2D6 diplotype** | **CYP2D6 activity score** | **CYP2D6 predicted phenotype** | **CYP3A4 diplotype** | **CYP3A5 diplotype** |
| --- | --- | --- | --- | --- | --- | --- | --- | --- | --- | --- | --- | --- | --- | --- | --- |
| 1 | control | *1A/*1A | *1/*1 | *1/*6 | IM | *1/*1 | NM | *2/*17 | 1.75 | IM | *1/*1 | 2 | NM | *1/*1 | *3/*3 |
| 2 | control | *1F/*1A | *1/*1 | *1/*6 | IM | *1/*1 | NM | *1/*17 | 2.75 | RM | *1/*1 | 2 | NM | *1/*1 | *3/*3 |
| 3 | control | NA | NA | *1/*6 | IM | *1/*2 | IM | *1/*1 | 2 | NM | *1/*1 | 2 | NM | *1/*1 | *3/*3 |
| 4 | control | *1F/*1J | *1/*1 | *1/*1 | NM | *1/*1 | NM | *1/*17 | 2.75 | RM | *1/*1 | 2 | NM | *1/*1 | *3/*3 |
| 5 | control | *1F/*1A | *1/*1 | *4/*6 | IM | *1/*1 | NM | *2/*2 | 0 | PM | *4/*4 | 0 | PM | *1/*1 | *3/*3 |
| 6 | control | *1A/*1A | *1/*1 | *6/*6 | PM | *1/*1 | NM | *2/*17 | 1.75 | IM | *1/*1x2 | 3 | UM | *1/*1 | *3/*3 |
| 7 | control | *1F/*1A | *1/*1 | *1/*1 | NM | *1/*1 | NM | *1/*2 | 1 | IM | *4/*4 | 0 | PM | *1/*1 | *3/*3 |
| 8 | control | *1F/*1A | *1/*14 | *1/*6 | IM | *1/*3 | IM | *1/*17 | 2.75 | RM | *1/*6 | 1 | IM | *1/*1 | *3/*3 |
| 9 | control | *1F/*1J | *1/*1 | *1/*6 | IM | *2/*3 | PM | *1/*1 | 2 | NM | *4/*4 | 0 | PM | *1/*1 | *3/*3 |
| 10 | control | *1F/*1F | *1/*1 | *1/*6 | IM | *1/*1 | NM | *1/*17 | 2.75 | RM | *1/*41 | 1.25 | NM | *1/*1 | *3/*3 |
| 11 | HELPcB | *1F/*1F | *1/*1 | *1/*5 | NM | *1/*2 | IM | *1/*17 | 2.75 | RM | *1/*1 | 2 | NM | *1/*22 | *3/*3 |
| 12 | HELPcB | *1F/*1A | *1/*1 | *6/*6 | PM | *1/*1 | NM | *1/*2 | 1 | IM | *1/*1 | 2 | NM | *1/*1 | *3/*3 |
| 13 | HELPcB | *1F/*1A | *1/*9 | *1/*22 | RM | *1/*2 | IM | *1/*1 | 2 | NM | *1/*1 | 2 | NM | *1/*1 | *3/*3 |
| 14 | HELPcB | *1F/*1A | *1/*1 | *1/*6 | IM | *1/*1 | NM | *1/*17 | 2.75 | RM | *1/*4 | 1 | IM | *1/*1 | *3/*3 |
| 15 | HELPcB | *1F/*1A | *1/*1 | *1/*5 | NM | *1/*3 | IM | *1/*17 | 2.75 | RM | *1/*9 | 1.25 | NM | *1/*1 | *1/*3 |
| 16 | HELPcB | *1F/*1A | *1/*1 | *1/*1 | NM | *1/*2 | IM | *1/*1 | 2 | NM | *9/*9 | 0.5 | IM | *1/*1 | *3/*3 |
| 17 | HELPcB | *1F/*1A | *1/*1 | *1/*5 | NM | *1/*1 | NM | *1/*17 | 2.75 | RM | *1/*4 | 1 | IM | *1/*22 | *3/*3 |
| 18 | HELPcB | *1F/*1A | *1/*1 | *1/*5 | NM | *1/*1 | NM | *1/*1 | 2 | NM | *1/*1x2 | 3 | UM | *1/*1 | *1/*3 |
| 19 | HELPcB | *1F/*1A | *1/*1 | *5/*6 | IM | *1/*1 | NM | *1/*17 | 2.75 | RM | *1/*1 | 2 | NM | *1/*1 | *3/*3 |
| 20 | HELPcB | *1F/*1A | *1/*1 | *1/*6 | IM | *1/*2 | IM | *1/*17 | 2.75 | RM | *1/*10 | 1.25 | NM | *1/*1 | *1/*3 |

Supplemental table 3: Genotyping results of the study participants sorted into control and HELPcB group.

The diplotype of the respective CYP enzymes, the genotype-predicted metabolizer phenotype (CYP2B6, CYP2C9, CYP2C19 and CYP2D6) and the activity score (CYP2C19 and CYP2D6) are shown. PM: poor metabolizer, IM: intermediate metabolizer, NM: normal metabolizer, RM: rapid metabolizer UM: ultrarapid metabolizer


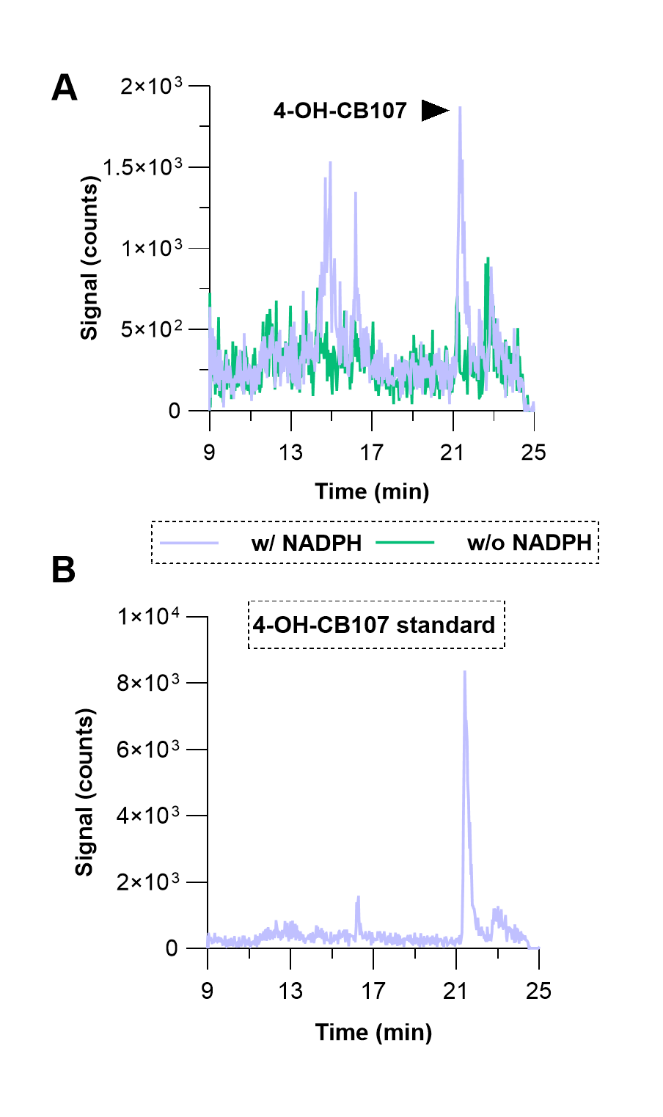


Supplemental figure 1: Chromatogram of the 4-OH-CB107 reference standard in comparison to the biocatalysis of PCB118 with recombinant CYP1A2. A: chromatogram of an incubation of PCB118 with recombinant CYP1A2 with and without NADPH shown in Fig. 4H. B: 4-OH-CB107 reference standard.

**Materials**

Caffeine (C0750, Sigma-Aldrich), paraxanthine (D5385, Sigma-Aldrich), midazolam (M908, Sigma-Aldrich), 1-hydroxylmidazolam (Cay10385-5, Cayman Chemical) metoprolol (HY-17503, MedChemExpress), α-hydroxymetoprolol (Cay28020-1, Cayman Chemical), torsemide (HY-B0247, MedChemExpress), hydroxytorsemide (sc-394035, Santa Cruz Biotech), omeprazole (HY-B0113, MedChemExpress), 5-hydroxyomeprazole (Cay10009028-500, Cayman Chemical), efavirenz (sc-207612, Santa Cruz Biotech), rac 8-hydroxyefavirenz (sc-208270, Santa Cruz Biotech), caffeine-d9 (725625-100MG, Sigma-Aldrich), paraxanthine-d6 (Cay9003564-1, Cayman Chemical), midazolam-d4 (M-918, Merck), 1-hydroxymidazolam-d4 (H-921, Merck), metoprolol-d6 tartrate (Cay28188-1, Cayman Chemical), α-hydroxymetoprolol-d5 (TOR-H948392-1MG, Biozol), torsemide-d7 (TOR-T548752-1MG, Biozol), hydroxytorsemide-d7 (TOR-H969982-1MG, Biozol), omeprazole-d3 (CMS-CS-4014-1MG, Biozol) , 5-hydroxyomeprazol-d3 sodium salt (TOR-H948864-1MG, Biozol), rac efavirenz-d5 (sc-219826, Santa Cruz Biotech), rac 8-hydroxyefavirenz-d4 (sc-219795, Santa Cruz Biotech), β-glucuronidase (347420-1MU, Sigma-Aldrich), methanol (HPLC gradient grade, 8402.2500, J.T. Baker), water (LiChrosolv LC-MS grade, MC1153332500, Merck), formic acid (HiPersolv CHROMANORM LC-MS grade, 84.865.180, VWR), bovine serum (2203-010, Acila), dimethyl sulfoxide (DMSO; A994.2, ROTH)

**Methods**

Calibration curves consisted of a minimum of 6 non-zero standards and a minimum of 4 quality control concentration levels for each analyte. The lowest concentration QC represents the lower limit of quantification (LLOQ).

Calibration curves were constructed with linear regression and a weighting of 1/x^2^. The linear calibration range for the analytes given in ng/ml was as follows: caffeine: 10 🡪 4000, paraxanthine: 10 🡪 4000, midazolam: 0.05 🡪 40, 1-hydroxymidazolam: 0.2 🡪 80, metoprolol: 0.25 🡪 200, α-hydroxymetoprolol: 0.25 🡪 200, Omeprazole: 0.25 🡪 200 (increased to 800 during sample measurement), 5-hydroxyomeprazole: 0.25 🡪 200, torsemide: 1 🡪 800 (elongated to 0.5 in glucuronidase dataset), OH-torsemide 1 🡪 800 (elongated to 0.5 in glucuronidase dataset), efavirenz: 0.625 🡪 500, 8-OH-efavirenz: 0.25 🡪 200.


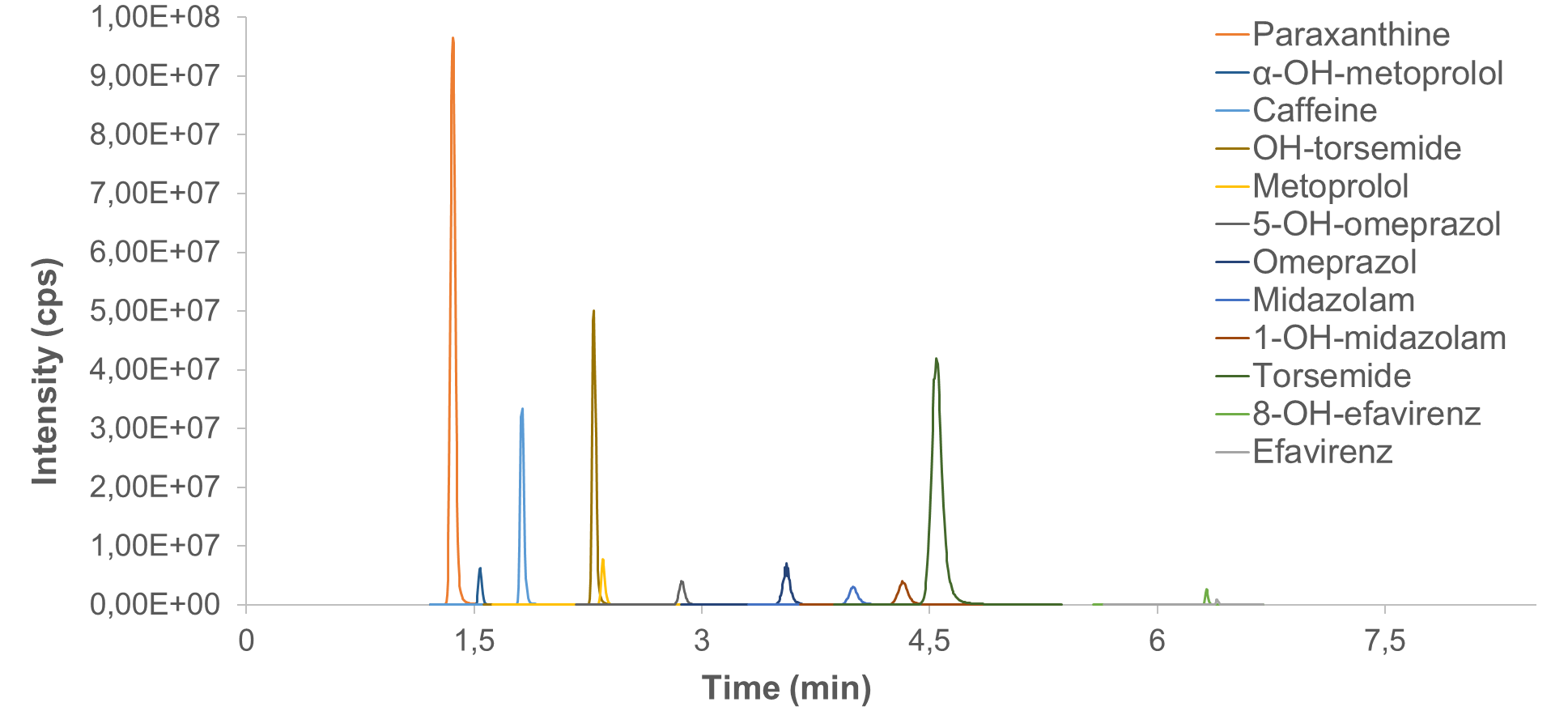


Supplemental figure 2: Representative chromatogram of all substrates and metabolites of the cocktail from the highest calibrator sample in bovine serum is shown. For clarity, only the quantifier MRM transitions are shown.

Supplemental table 4: Mass spectrometer settings for the analytes. Given are the MRM transitions with Q1 and Q3 masses (*m/z*), polarity, declustering potential (DP), collision energy (CE) and the retention time (RT).

| **Analayte** |  | **Q1 mass (m/z)** | **Q3 mass (m/z)** | **Polarity** | **DP (V)** | **CE (V)** | **RT (min)** |
| --- | --- | --- | --- | --- | --- | --- | --- |
| Caffeine | quant | 195 | 138 | + | 96 | 35 | 1,85 |
|  | qual | 195 | 110 | + | 81 | 31 |  |
| Caffeine-d9 | IS | 204 | 144 | + | 71 | 29 |  |
| Paraxanthine | quant | 181 | 124 | + | 46 | 27 | 1,40 |
|  | qual | 181 | 96 | + | 46 | 31 |  |
| Paraxanthine-d6 | IS | 187 | 127 | + | 80 | 29 |  |
| Midazolam | quant | 326 | 291 | + | 41 | 37 | 4,05 |
|  | qual | 326 | 249 | + | 41 | 31 |  |
| Midazolam-d4 | IS | 330 | 295 | + | 111 | 37 |  |
| 1-hydroxymidazolam | quant | 342 | 203 | + | 66 | 35 | 4,40 |
|  | qual | 342 | 324 | + | 66 | 29 |  |
| Hydroxymidazolam-d4 | IS | 346 | 203 | + | 71 | 37 |  |
| Metoprolol | quant | 268 | 116 | + | 46 | 25 | 2,37 |
|  | qual | 268 | 121 | + | 46 | 31 |  |
| Metoprolol-d6 | IS | 274 | 122 | + | 80 | 27 |  |
| α-hydroxymetoprolol | quant | 284 | 116 | + | 66 | 25 | 1,56 |
|  | qual | 284 | 56 | + | 66 | 55 |  |
| α-hydroxymetoprolol-d5 | IS | 289 | 121 | + | 91 | 27 |  |
| Torsemide | quant | 349 | 264 | + | 36 | 23 | 4,62 |
|  | qual | 349 | 168 | + | 36 | 61 |  |
| Torsemide-d7 | IS | 356 | 264 | + | 26 | 25 |  |
| Hydroxytorsemide | quant | 365 | 280 | + | 56 | 23 | 2,31 |
|  | qual | 365 | 306 | + | 56 | 19 |  |
| Hydroxytorsemide-d7 | IS | 372 | 280 | + | 21 | 23 |  |
| Omeprazol | quant | 346 | 198 | + | 31 | 15 | 3,61 |
|  | qual | 346 | 136 | + | 31 | 49 |  |
| Omeprazol-d3 | IS | 349 | 198 | + | 60 | 15 |  |
| 5-hydroxyomeprazol | quant | 362 | 214 | + | 50 | 15 | 2,92 |
|  | qual | 362 | 152 | + | 50 | 47 |  |
| 5-hydroxyomeprazol-d3 | IS | 365 | 213 | + | 51 | 17 |  |
| Efavirenz | quant | 314 | 230 | - | 50 | 18 | 6,40 |
|  | qual | 314 | 244 | - | 50 | 22 |  |
| Efavirenz-d5 | IS | 319 | 248 | - | 50 | 24 |  |
| 8-hydroxyefavirenz | quant | 330 | 258 | - | 50 | 24 | 6,33 |
|  | qual | 330 | 210 | - | 50 | 24 |  |
| 8-hydroxyefavirenz-d4 | IS | 334 | 258 | - | 50 | 22 |  |

CE: collision energy, DP: declustering potential, RT: retention time, Q1: first quadrupole, Q3: third quadrupole, quant: quantifier, qual: qualifier, IS: internal standard

Supplemental table 5: Intra- and Interday Accuracy and Precision. For intraday accuracy and precision n=5 quality control samples were measured against a calibration curve injected in duplicate. For interday accuracy and precision, three sets of calibration curves and quality control samples were prepared separately on three different days.

|  |  | Intraday (n=5) | | Interday (n=3) | |
| --- | --- | --- | --- | --- | --- |
| **Analyte** | **Conc. (ng/ml)** | **CV (%)** | **Av. Accuracy (%)** | **CV (%)** | **Av. Accuracy (%)** |
| **Caffeine** | 10,00 | 2,09 | 95,1 | 13,84 | 89,56 |
|  | 30,00 | 17,4 | 109,99 | 3,68 | 104,80 |
|  | 1200,00 | 2,08 | 100,73 | 5,38 | 107,25 |
|  | 3000,00 | 1,79 | 91,65 | 3,16 | 97,93 |
| **Paraxanthine** | 10,00 | 1,07 | 93,56 | 3,38 | 94,78 |
|  | 30,00 | 15,14 | 106,59 | 1,07 | 102,34 |
|  | 1200,00 | 1,10 | 95,83 | 0,71 | 100,39 |
|  | 3000,00 | 1,38 | 88,04 | 0,75 | 91,78 |
| **Midazolam** | 0,05 | 7,69 | 108,95 | 5,31 | 90,71 |
|  | 0,10 | 6,59 | 102,56 | 5,30 | 105,44 |
|  | 0,30 | 3,64 | 99,9 | 2,42 | 99,81 |
|  | 12,00 | 2,13 | 99,7 | 1,95 | 103,17 |
|  | 30,00 | 2,32 | 95,77 | 2,10 | 99,29 |
| **1-OH-midazolam** | 0,20 | 17,89 | 95,03 | 15,17 | 94,90 |
|  | 0,60 | 3,28 | 106,3 | 6,21 | 101,42 |
|  | 24,00 | 1,93 | 103,75 | 3,86 | 110,94 |
|  | 60,00 | 1,41 | 97,82 | 3,26 | 102,39 |
| **Metoprolol** | 0,25 | 2,34 | 95,18 | 7,80 | 101,52 |
|  | 0,50 | 9,82 | 101,36 | 3,91 | 111,16 |
|  | 1,50 | 5,23 | 100,15 | 2,66 | 106,53 |
|  | 60,00 | 3,8 | 99,34 | 2,02 | 103,39 |
|  | 150,00 | 2,63 | 97,76 | 0,83 | 98,60 |
| **α-OH-metoprolol** | 0,25 | 7,75 | 87,89 | 6,81 | 78,64 |
|  | 0,50 | 5,39 | 93,42 | 5,23 | 89,42 |
|  | 1,50 | 5,74 | 91,74 | 2,49 | 92,82 |
|  | 60,00 | 2,32 | 86,72 | 0,89 | 89,21 |
|  | 150,00 | 1,14 | 82,4 | 2,28 | 84,18 |
| **Torsemide** | 1,00 | 0,89 | 98,12 | 3,59 | 99,31 |
|  | 2,00 | 0,85 | 102,32 | 2,99 | 106,42 |
|  | 6,00 | 2,33 | 100,76 | 0,57 | 105,26 |
|  | 240,00 | 2,26 | 99,79 | 0,63 | 104,67 |
|  | 600,00 | 1,21 | 93,4 | 0,63 | 96,57 |
| **OH-torsemide** | 1,00 | 4,94 | 93,43 | 4,44 | 100,75 |
|  | 2,00 | 2,73 | 101,13 | 3,97 | 105,40 |
|  | 6,00 | 1,26 | 100,83 | 1,06 | 108,96 |
|  | 240,00 | 4,58 | 97,79 | 1,48 | 104,50 |
|  | 600,00 | 1,1 | 92,37 | 0,20 | 95,48 |
| **Omeprazole** | 0,25 | 5,41 | 107,84 | 7,50 | 92,32 |
|  | 0,50 | 4,2 | 106,66 | 5,24 | 103,54 |
|  | 1,50 | 7,82 | 95,84 | 4,79 | 106,44 |
|  | 60,00 | 8,06 | 99,46 | 1,90 | 107,91 |
|  | 150,00 | 2,38 | 95,84 | 4,50 | 100,76 |
| **5-OH-omeprazole** | 0,25 | 9,32 | 91,34 | 15,55 | 96,79 |
|  | 0,50 | 10,55 | 98,69 | 4,77 | 97,84 |
|  | 1,50 | 4,6 | 87,92 | 5,59 | 92,73 |
|  | 60,00 | 4,42 | 95,13 | 4,64 | 99,06 |
|  | 150,00 | 4,63 | 85,66 | 5,73 | 94,47 |
| **Efavirenz** | 0,63 | 10,21 | 104,47 | 18,87 | 114,07 |
|  | 1,25 | 5,77 | 110,97 | 10,26 | 120,59 |
|  | 3,75 | 3,49 | 100,36 | 3,58 | 109,08 |
|  | 150,00 | 3,18 | 105,87 | 1,23 | 106,33 |
|  | 375,00 | 2,84 | 99,34 | 2,19 | 100,57 |
| **8-OH-efavirenz** | 0,25 | 7,68 | 78,92 | 1,97 | 100,19 |
|  | 0,50 | 7,52 | 74,79 | 3,03 | 84,53 |
|  | 1,50 | 4,13 | 74,74 | 2,78 | 80,09 |
|  | 60,00 | 2,07 | 75,34 | 2,35 | 81,26 |
|  | 150,00 | 2,06 | 71,44 | 3,55 | 77,47 |

CV: coefficient of variation, Av. accuracy: average accuracy, conc: concentration

Supplemental table 6: Stability of the substrates and metabolites. Quality control samples were left at room temperature for 19 hours (benchtop stability) and at 10° C for 24 hours in the autosampler (autosampler stability) or were measured after three freeze-thaw cycles. Stability samples were measured against a freshly prepared set of calibrators and quality control samples.

|  |  | Autosampler stability (24 h) | | Benchtop stability (19 h) | | 3x freeze-thaw | |
| --- | --- | --- | --- | --- | --- | --- | --- |
| Analyte | Conc. (ng/ml) | CV (%) | Av. Accuracy (%) | CV (%) | Ac. Accuracy (%) | CV (%) | Av. Accuracy (%) |
| Caffeine | 10 | 2,76 | 92,31 | 6,13 | 94,63 | 11,6 | 107,62 |
|  | 30 | 3,39 | 107,31 | 1,39 | 107,73 | 0,94 | 107,18 |
|  | 1200 | 0,83 | 109,14 | 1,1 | 107,12 | 2,44 | 105,12 |
|  | 3000 | 0,69 | 97,17 | 5,68 | 97,25 | 0,24 | 97,54 |
| Paraxanthine | 10 | 4,62 | 96,28 | 3,41 | 89,99 | 1,43 | 96,57 |
|  | 30 | 0,27 | 105,74 | 3,2 | 105,38 | 0,89 | 103,75 |
|  | 1200 | 1,38 | 105,06 | 1,25 | 103,5 | 3,06 | 100,61 |
|  | 3000 | 1,1 | 93,91 | 5,21 | 94,2 | 0,81 | 92,32 |
| Midazolam | 0,05 | 12,03 | 103,51 | 9,92 | 89,44 | 14,24 | 109,93 |
|  | 0,1 | 11,28 | 107,46 | 8,52 | 106,81 | 5,37 | 104,96 |
|  | 0,3 | 7,46 | 104,87 | 1,36 | 104,39 | 4,87 | 98,61 |
|  | 12 | 0,36 | 110,93 | 1,2 | 109,38 | 2,49 | 103,85 |
|  | 30 | 2,77 | 99,76 | 4,72 | 99,8 | 1,86 | 97,41 |
| 1-OH-midazolam | 0,2 | 11,27 | 102,59 | 8,55 | 82,15 | 6,31 | 108,6 |
|  | 0,6 | 7,06 | 107,25 | 1,5 | 102,06 | 4,96 | 109,36 |
|  | 24 | 0,85 | 113,74 | 1,12 | 112,46 | 3,49 | 106,45 |
|  | 60 | 1,07 | 105,87 | 3,29 | 105,11 | 1,16 | 100,47 |
| Metoprolol | 0,25 | 8,96 | 105,01 | 13,13 | 92,77 | 2,67 | 99,7 |
|  | 0,5 | 3,6 | 105,36 | 6,2 | 106,29 | 2,68 | 109,56 |
|  | 1,5 | 2,57 | 112,83 | 3,89 | 111,4 | 2,93 | 103,63 |
|  | 60 | 0,91 | 111,39 | 1,42 | 110,41 | 3,31 | 105,26 |
|  | 150 | 3,32 | 100,37 | 4,53 | 102,76 | 0,74 | 98,84 |
| OH-metoprolol | 0,25 | 4,88 | 90,24 | 11,29 | 81,14 | 12,58 | 85,17 |
|  | 0,5 | 2,6 | 101,05 | 2,21 | 90,1 | 8,42 | 95,27 |
|  | 1,5 | 4,38 | 96,56 | 4,92 | 94,57 | 1,46 | 95,33 |
|  | 60 | 3,78 | 97,01 | 2,7 | 95,47 | 3,04 | 91,98 |
|  | 150 | 3,77 | 88,29 | 6 | 88,26 | 1,69 | 87,96 |
| Torsemide | 1 | 1,75 | 105,13 | 16,81 | 97,46 | 4,41 | 102,74 |
|  | 2 | 4,66 | 108,32 | 1,72 | 107,17 | 0,72 | 105,85 |
|  | 6 | 1,21 | 108,92 | 0,89 | 107,51 | 1,16 | 106,12 |
|  | 240 | 1,6 | 109,92 | 0,64 | 108,15 | 3,67 | 103,96 |
|  | 600 | 0,86 | 97,91 | 5,03 | 99,27 | 1,16 | 96,67 |
| OH-torsemide | 1 | 1,05 | 109,17 | 16,65 | 106,91 | 3,36 | 105,26 |
|  | 2 | 1,35 | 113,11 | 2,49 | 107,98 | 3,12 | 105,85 |
|  | 6 | 0,24 | 109,32 | 3,01 | 111,3 | 1,28 | 107,78 |
|  | 240 | 0,65 | 108,82 | 0,48 | 106,75 | 2,87 | 104,11 |
|  | 600 | 0,79 | 99,55 | 5,9 | 99,14 | 0,49 | 96,28 |
| Omeprazole | 0,25 | 8,36 | 102,23 | 9,85 | 91,86 | 5,51 | 112,67 |
|  | 0,5 | 10,96 | 114,94 | 1,85 | 117,42 | 3,3 | 112,03 |
|  | 1,5 | 5,48 | 114,49 | 4,66 | 115,14 | 5,97 | 111,42 |
|  | 60 | 1,89 | 111,22 | 1,77 | 112,32 | 4,67 | 106,21 |
|  | 150 | 4,13 | 103,07 | 3,29 | 104,38 | 4,08 | 105,55 |
| 5-OH-omeprazole | 0,25 | 9,04 | 90,94 | 14,97 | 99,1 | 9,16 | 101,98 |
|  | 0,5 | 9,43 | 112,52 | 8,89 | 95,94 | 8,78 | 105,73 |
|  | 1,5 | 4,72 | 102,58 | 5,99 | 98,98 | 3,7 | 103,03 |
|  | 60 | 4,99 | 104,61 | 1,72 | 99,68 | 3,96 | 99,23 |
|  | 150 | 0,62 | 97,73 | 2,82 | 92,79 | 3,23 | 100,57 |
| Efavirenz | 0,63 | 7,89 | 109,58 | 19,5 | 92,21 | 2,91 | 124,41 |
|  | 1,25 | 4,73 | 114,46 | 10,8 | 102,48 | 10,05 | 117,78 |
|  | 3,75 | 5,36 | 114,56 | 0,77 | 107,06 | 4,73 | 111,15 |
|  | 150 | 2,08 | 115,3 | 2,26 | 111,5 | 5,41 | 112,08 |
|  | 375 | 1,59 | 106,16 | 4,61 | 104,9 | 0,56 | 107,17 |
| 8-OH-efavirenz | 0,25 | 8,8 | 86,81 | 29,23 | 69,5 | 3,77 | 84,61 |
|  | 0,5 | 7,51 | 86,73 | 4,57 | 74,06 | 4,53 | 84,9 |
|  | 1,5 | 3,16 | 88,49 | 0,63 | 76,61 | 2,09 | 79,73 |
|  | 60 | 2,36 | 86,15 | 2,23 | 77,52 | 3,57 | 79,98 |
|  | 150 | 2,57 | 78,85 | 6,95 | 74,14 | 0,86 | 77,49 |

CV: coefficient of variation, Av. accuracy: average accuracy, conc: concentration, RT: room temperature

Supplemental table 7: Assessment of the matrix effect. Analytes and internal standards were spiked in 4 different matrices at two different concentration levels (low QC and high QC). The matrix factor was calculated by comparing with the detector signal of analytes in pure solvent samples. Matrix effect was considered acceptable for an analyte and concentration level with a % CV below 15.

|  | **low QC** | | **High QC** | |
| --- | --- | --- | --- | --- |
|  | CV % | ng/ml | CV % | ng/ml |
| Midazolam | 1,68 | 0,3 | 3,40 | 30 |
| 1-OH-midazolam | 3,59 | 0,6 | 1,53 | 60 |
| Metoprolol | 6,10 | 1,5 | 1,97 | 150 |
| α-OH-metoprolol | 10,34 | 1,5 | 5,34 | 150 |
| Torsemide | 8,55 | 6 | 2,05 | 600 |
| OH-torsemide | 13,17 | 6 | 3,37 | 600 |
| Omeprazole | 6,01 | 1,5 | 7,21 | 150 |
| 5-OH-omeprazole | 8,30 | 1,5 | 2,77 | 150 |
| Efavirenz | 9,02 | 3,75 | 2,37 | 375 |
| 8-OH-efavirenz | 4,21 | 1,5 | 5,06 | 150 |

CV: coefficient of variation, QC: quality control

Supplemental figure 3: Assessment of the matrix effect for caffeine and paraxanthine. Due to high background levels of caffeine and paraxanthine in human plasma samples, the slope of the calibration curves in bovine serum and methanol were compared. The slopes for caffeine and paraxanthine in methanol and bovine serum were similar with 0.00089 and 0.00086, and 0.0029 and 0.0027, respectively.

Supplemental table 8: Accuracy and precision over all study batches (with and without glucuronidase treatment). Accuracy (%) and precision (CV %) were within ± 15 % for all quality control levels and analytes except for 0.5 ng/ml QC for α-OH-metoprolol and 8-OH-efavirenz. The quantification range for torsemide and OH-torsemide was decreased to 0.5 ng/ml. A set of calibrators and quality controls was processed for each batch and injected in front and after study samples.

|  |  | native dataset | | glucuronidase dataset | |
| --- | --- | --- | --- | --- | --- |
|  | **Conc. (ng/ml)** | **Av. Accuracy (%)** | **CV (%)** | **Av. Accuracy (%)** | **CV (%)** |
| Caffeine | 10,00 | 94,68 | 7,72 | 96,34 | 14,45 |
|  | 30,00 | 102,09 | 6,57 | 99,98 | 3,89 |
|  | 1200,00 | 102,79 | 2,14 | 101,88 | 5,44 |
|  | 3000,00 | 98,84 | 4,12 | 96,58 | 1,92 |
| Paraxanthine | 10,00 | 89,92 | 7,56 | 93,50 | 9,38 |
|  | 30,00 | 98,62 | 4,54 | 98,99 | 2,58 |
|  | 1200,00 | 99,55 | 1,76 | 97,59 | 5,11 |
|  | 3000,00 | 94,09 | 4,06 | 91,43 | 1,34 |
| Midazolam | 0,05 | 95,94 | 6,84 | 95,74 | 13,20 |
|  | 0,10 | 90,59 | 9,17 | 88,55 | 7,36 |
|  | 0,30 | 95,32 | 4,79 | 96,24 | 4,58 |
|  | 12,00 | 102,70 | 2,98 | 101,72 | 5,48 |
|  | 30,00 | 102,26 | 4,73 | 101,80 | 2,33 |
| 1-OH-midazolam | 0,20 | 97,18 | 7,06 | 97,58 | 12,18 |
|  | 0,60 | 98,88 | 6,18 | 102,33 | 5,18 |
|  | 24,00 | 107,09 | 3,24 | 105,08 | 5,42 |
|  | 60,00 | 105,11 | 4,15 | 104,89 | 2,69 |
| Metoprolol | 0,25 | 98,56 | 6,57 | 97,65 | 12,42 |
|  | 0,5 | 91,45 | 6,52 | 90,75 | 8,40 |
|  | 1,5 | 98,19 | 4,58 | 98,08 | 3,45 |
|  | 60 | 104,82 | 1,76 | 103,21 | 5,52 |
|  | 150 | 104,09 | 4,84 | 102,83 | 2,33 |
| α-OH-metoprolol | 0,25 | 86,67 | 7,94 | 91,16 | 9,93 |
|  | 0,50 | 82,28 | 7,82 | 81,38 | 6,16 |
|  | 1,50 | 88,28 | 4,25 | 88,35 | 4,18 |
|  | 60,00 | 91,09 | 2,32 | 90,30 | 6,28 |
|  | 150,00 | 90,35 | 5,31 | 88,95 | 2,24 |
| Torsemide | 0,5 | - | - | 96,26 | 4,68 |
|  | 1 | 97,57 | 3,40 | 97,20 | 3,35 |
|  | 2 | 91,53 | 5,01 | 90,20 | 2,91 |
|  | 6 | 97,93 | 3,55 | 97,95 | 1,71 |
|  | 240 | 103,02 | 1,80 | 102,00 | 5,21 |
|  | 600 | 101,46 | 5,19 | 100,47 | 1,53 |
| OH-torsemide | 0,5 | - | - | 96,43 | 7,03 |
|  | 1 | 96,53 | 4,74 | 97,71 | 3,53 |
|  | 2 | 91,05 | 5,84 | 90,58 | 4,07 |
|  | 6 | 98,88 | 4,32 | 100,19 | 3,39 |
|  | 240 | 103,81 | 2,92 | 102,65 | 5,39 |
|  | 600 | 100,29 | 4,85 | 98,95 | 2,50 |
| Omeprazole | 0,25 | 105,85 | 7,62 | 103,32 | 9,59 |
|  | 0,5 | 94,83 | 4,47 | 95,74 | 7,15 |
|  | 1,5 | 103,90 | 4,87 | 103,53 | 6,71 |
|  | 60 | 110,26 | 2,60 | 106,89 | 6,62 |
|  | 150 | 109,03 | 4,40 | 107,12 | 7,16 |
|  | 600 | 96,36 | 3,10 | 95,45 | 5,12 |
| 5-OH-omeprazole | 0,25 | 99,88 | 8,55 | 100,33 | 12,79 |
|  | 0,5 | 85,37 | 6,55 | 89,61 | 8,72 |
|  | 1,5 | 92,05 | 5,98 | 92,78 | 5,78 |
|  | 60 | 97,00 | 3,34 | 98,08 | 6,34 |
|  | 150 | 97,31 | 4,05 | 98,25 | 5,30 |
| Efavirenz | 0,63 | 102,02 | 11,43 | 98,35 | 11,53 |
|  | 1,25 | 95,67 | 13,23 | 93,29 | 8,57 |
|  | 3,75 | 102,65 | 5,09 | 100,40 | 5,34 |
|  | 150 | 106,23 | 3,10 | 103,47 | 5,88 |
|  | 375 | 106,66 | 5,39 | 105,65 | 2,79 |
| 8-OH-efavirenz | 0,25 | 88,42 | 7,59 | 78,01 | 10,00 |
|  | 0,5 | 73,77 | 7,08 | 67,96 | 6,48 |
|  | 1,5 | 75,50 | 3,44 | 73,41 | 2,71 |
|  | 60 | 77,52 | 2,34 | 76,54 | 5,02 |
|  | 150 | 78,21 | 4,58 | 76,69 | 2,93 |

CV: coefficient of variation, Av. accuracy: average accuracy, conc: concentration
